# Spatial transcriptomics delineates molecular features and cellular plasticity in lung adenocarcinoma progression

**DOI:** 10.1038/s41421-023-00591-7

**Published:** 2023-09-19

**Authors:** Yan Wang, Bing Liu, Qingjie Min, Xin Yang, Shi Yan, Yuanyuan Ma, Shaolei Li, Jiawen Fan, Yaqi Wang, Bin Dong, Huajing Teng, Dongmei Lin, Qimin Zhan, Nan Wu

**Affiliations:** 1 Key Laboratory of Carcinogenesis and Translational Research (Ministry of Education/Beijing), Laboratory of Molecular Oncology, Peking University Cancer Hospital & Institute, Beijing, China; 2 Key Laboratory of Carcinogenesis and Translational Research (Ministry of Education), Department of Thoracic Surgery II, Peking University Cancer Hospital & Institute, Beijing, China; 3 Key Laboratory of Carcinogenesis and Translational Research (Ministry of Education), Department of Pathology, Peking University Cancer Hospital & Institute, Beijing, China; 4 Key Laboratory of Carcinogenesis and Translational Research (Ministry of Education/Beijing), Central Laboratory, Peking University Cancer Hospital and Institute, Beijing, China; 5 Key Laboratory of Carcinogenesis and Translational Research (Ministry of Education/Beijing), Department of Radiation Oncology, Peking University Cancer Hospital & Institute, Beijing, China; 6 State Key Laboratory of Molecular Oncology, Peking University Cancer Hospital & Institute, Beijing, China; 7 Cancer Institute, Peking University Shenzhen Hospital, Shenzhen Peking University-Hong Kong University of Science and Technology (PKU-HKUST) Medical Center, Shenzhen, Guangdong China; 8 Research Unit of Molecular Cancer Research, Chinese Academy of Medical Sciences, Beijing, China; 9 International Cancer Institute, Peking University Health Science Center, Beijing, China; 10 Soochow University Cancer institute, Suzhou, Jiangsu China

**Keywords:** Cancer genomics, Non-small-cell lung cancer

## Abstract

Indolent (lepidic) and aggressive (micropapillary, solid, and poorly differentiated acinar) histologic subtypes often coexist within a tumor tissue of lung adenocarcinoma (LUAD), but the molecular features associated with different subtypes and their transitions remain elusive. Here, we combine spatial transcriptomics and multiplex immunohistochemistry to elucidate molecular characteristics and cellular plasticity of distinct histologic subtypes of LUAD. We delineate transcriptional reprogramming and dynamic cell signaling that determine subtype progression, especially hypoxia-induced regulatory network. Different histologic subtypes exhibit heterogeneity in dedifferentiation states. Additionally, our results show that macrophages are the most abundant cell type in LUAD, and identify different tumor-associated macrophage subpopulations that are unique to each histologic subtype, which might contribute to an immunosuppressive microenvironment. Our results provide a systematic landscape of molecular profiles that drive LUAD subtype progression, and demonstrate potentially novel therapeutic strategies and targets for invasive lung adenocarcinoma.

## Introduction

Tumor progression is characterized by dynamic molecular and phenotypic changes of tumor cells, referred to as cellular plasticity, and is associated with dedifferentiated states concomitant with therapy resistance and poor clinical outcomes^1^. Intrinsic and extrinsic factors, such as genetic alterations, epigenetic modifications, transcriptional changes, and treatment-induced selective pressures sculpt cancer cell plasticity, therefore contributing to inter- and intra-tumor heterogeneities^2^. Cell fate transitions, including epithelial-to-mesenchymal transition (EMT) and mesenchymal-to-epithelial transition (MET), are multi-faceted and fundamental processes in cell reprogramming and tumor metastasis^3,4^. Cellular plasticity is usually related to a poorly differentiated phenotype^5^, which can be mediated by transcription factors and microRNAs that modulate cell polarity, adhesion and motility^6^.

Lung adenocarcinoma (LUAD), one of the most prevalent and lethal malignancies, is prominently characterized by histologic heterogeneity, as well as cellular and molecular heterogeneities^7,8^. Multiple histologic subtypes often coexist in a tumor mass. Some of these subtypes (micropapillary, solid and complex glandular) are highly invasive, but others (lepidic) show indolent growth^9^. Although extensive genetic and epigenetic heterogeneities in LUAD have been demonstrated^10^, the molecular characteristics and biological interactions facilitating the transition of histologic subtypes during LUAD progression remain elusive.

Bulk RNA-seq homogenizes tissue context, and yields an averaged expression profile of diverse types of cells, such as epithelial, endothelial, stromal and immune cells, in a given tissue. The advent of single-cell RNA-sequencing (scRNA-seq) facilitates comprehensive profiling of transcriptional heterogeneity at single-cell resolution, and allows to decode intercellular signaling networks in tumor microenvironment (TME)^11–20^. For example, subsolid nodules of LUAD exhibit abundant cytotoxic nature killer/T cells that play a vital role in immunosurveillance^21^. Additionally, comprehensive characterizations of tumor infiltrating lymphocytes revealed the dynamics of functional states of T cells in non-small cell lung cancer^22^. Recently, a systematic investigation of lung tissue compartments revealed rare cell types in lung tumors^23^, which provides molecular insights into cell state transitions during tumor evolution and progression^24^. Nonetheless, spatial localization information of individual cells is lacking in scRNA-seq analyses, thus preventing a deeper understanding of in situ intercellular communications as well as spatial niches that orchestrate development and tissue homeostasis^25^. Decoding molecular underpinnings of subtype-specific tumor progress is critical for therapeutic implications, but largely unexplored for LUAD. Recent advances in spatially resolved transcriptomics, including in situ hybridization and spatial barcoding, can complement the limitations of scRNA-seq by charting unbiased transcriptomic maps of entire tissue sections^26^. In addition, multiplex Immunohistochemistry/Immunofluorescence (mIHC/IF) techniques, such as chromogenic, metal-based, fluorescence-based, DNA barcoding-based platforms, also provide new options to investigate spatial distribution of diverse cells via simultaneous detection of multiple markers in a tissue section^27^.

Here, by applying spatial transcriptomics (ST) and mIHC to primary invasive LUAD, we elucidate molecular features and TME driving cancer subtype progression. We investigate spatial cellular composition, cell signaling heterogeneity, dedifferentiation states, and immune landscape, especially spatial heterogeneity of macrophages from indolent to aggressive histologic subtypes. Furthermore, we validate our findings in a public cohort. Overall, these results provide molecular characteristics underlying subtype transitions in LUAD progression, and might provide new insights into novel therapeutic strategies.

## Results

### Spatial transcriptomics reveal intratumor heterogeneity of LUAD

**Fig. 1:**
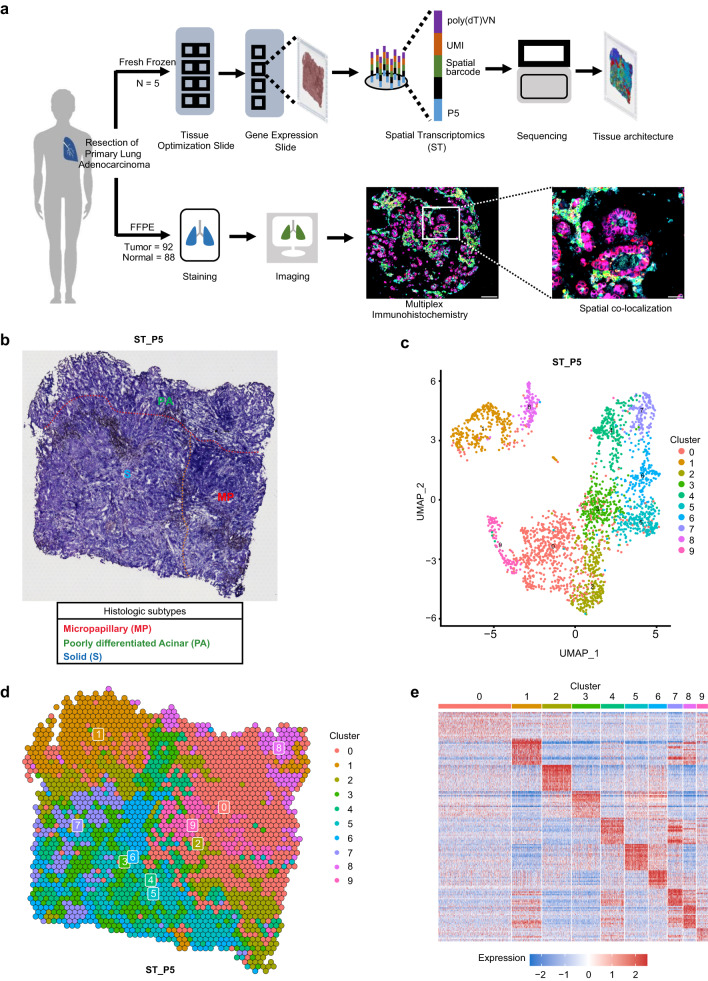
Histologic subtypes of LUAD revealed by ST. **a** Schematic representation of patient sample processing for ST and mIHC. **b** Annotated histologic subtypes for sample ST_P5, including micropapillary (red), poorly differentiated acinar (green), and solid (blue) subtypes. **c** UMAP plots of 2481 spatial spots (10 clusters). Spot color was consistent with corresponding cluster identity. **d** Spatial distribution and clustering of ST spots. **e** Heatmap showing DEGs of each cluster.

Based on histologic features, LUAD can be classified into five major subtypes, including lepidic (Lep), papillary (P), acinar (A), micropapillary (MP) and solid (S) subtypes, which is a predictor of recurrence and therapeutic resistance^9,28^. Most LUADs exhibit multiple histologic subtypes within a tissue. To elucidate cellular composition in different histologic subtypes, we performed ST on invasive LUAD samples from five patients (Supplementary Fig. S1) undergoing radical resection using 10× Visium platform (Fig. 1a; Supplementary Table S1). Transcriptomes from 18,475 spots were obtained with a median of 3690 genes (Supplementary Fig. S2) and 8178 unique molecular identifiers (Supplementary Table S2). Samples were annotated as distinct histologic subtypes (Fig. 1b) by thoracic pathologists. Clustering of transcriptional signatures of ST spots was determined by a shared nearest neighbor (SNN) modularity optimization, then Uniform Manifold Approximation and Projection (UMAP) was performed for dimensionality reduction^29^ (Fig. 1c; Supplementary Table S3). For example, ST_P5 tumor section that comprised MP, poorly differentiated acinar (PA) and S subtypes could be divided into ten clusters (Supplementary Fig. S3) based on differentially expressed genes (Supplementary Fig. S4 and Table S4), suggesting intra-tumor spatial heterogeneity (Fig. 1d, e).

**Fig. 2:**
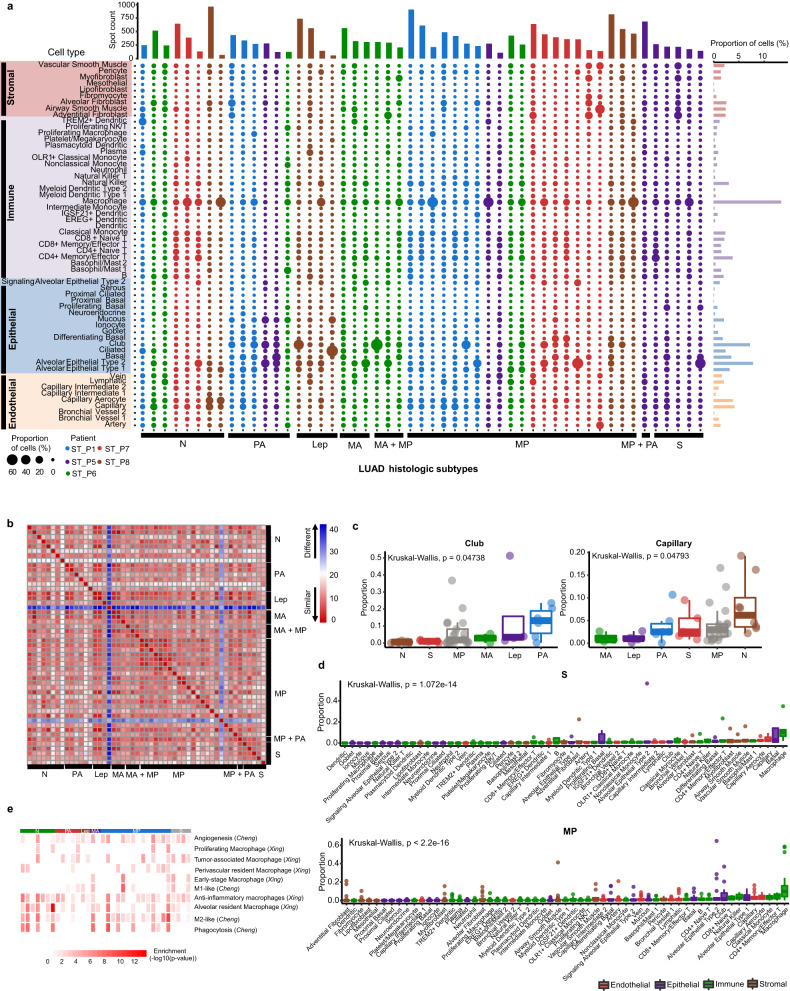
Identification and characterization of cellular composition across histologic subtypes. **a** Cell types composition inferred by molecular anchors transfer based on a comprehensive cell atlas of the human lung. The middle panel showed cell types (rows) by subtype-specific clusters (columns). The size of circle represents the average proportion of cell type in each cluster. The top histogram showing the number of spots covered in each cluster. The right histogram showing average fraction of each cell type in this cohort. **b** Similarity analysis of cellular composition across histologic subtypes using hamming distance between each pair of clusters. **c** Proportion differences of club cells and capillary cells among histologic subtypes. *P* values were calculated by Kruskal–Wallis rank sum test. Each dot represents a cluster. **d** Cellular compositions of S and MP subtypes. Kruskal–Wallis rank sum test. **e** Enrichment of phenotypic differences of macrophages using MIA based on two independent macrophage datasets^21,36^.

Using a comprehensive single-cell dataset of the human lung as a reference^23^, we performed anchors analysis (see Materials and methods) to estimate cell type composition and proportions of the mixture of cells within each spot (Supplementary Tables S3 and S5). Intriguingly, macrophages were the most abundant cell type in LUAD, accounting for 13.39% of all cells (Fig. 2a). Alveolar macrophages play an important role in the immune response to tissue infections, inflammation, and the maintenance of lung homeostasis^30^. The proportion of alveolar epithelial type II (AT2) (7.82%) in LUAD was larger than alveolar epithelial type I (AT1) (3.18%). AT2 cells secret pulmonary surfactant that reduces alveolar surface tension, and the dysfunction of surfactant leads to alveolar collapse^31^. We also observed a substantial number of club cells (7.2%), which were responsible for bronchiolar repair and regeneration^32^. Club cells drove tumorigenesis under environmental exposure to carcinogenic agents in adult mice, as described previously^33^. Furthermore, two capillary cell types, general capillary cells (vasomotor regulation and progenitor cells) and capillary aerocytes (gas exchange and leukocyte trafficking)^34^, were also abundant in each histologic subtype. Tumor vascularization, including capillary sprouting (commonly referred to as angiogenesis) and vessel co-option, is an important hallmark of neoplastic progression^35^. Alveolar capillary cells surrounding each alveolus form the respiratory surface for gas exchange, and play an important role in tumorigenesis and development. Additionally, the co-occurrence of several cell types was explored using pairwise cell-type correlation analysis. We observed significant spatial correlations within different cell types, for example, mucous vs. goblet cells, and CD4^+^ naive T cells vs. CD4^+^ memory/effector T cells (Supplementary Fig. S5).

Further similarity analysis of cellular composition across histologic subtypes indicated a high level of similarity in major cell types (Fig. 2b), except for two clusters (ST_P7 cluster 8 and ST_P8 cluster 8) (Supplementary Fig. S6a) which covered too few spots in the given slides (Supplementary Fig. S6b, c). Intriguingly, substantial changes of cellular proportion in different histologic subtypes were observed. The proportion of club cells was highest in PA subtype, whereas general capillary cells are the most abundant cells in normal lung tissue (Fig. 2c). In contrast, there are no differences in capillary aerocytes, AT2 cells and alveolar fibroblasts across histologic subtypes (Supplementary Fig. S7). Notably, macrophages accounted for the largest proportion of all cell types in S and MP subtypes, with an average of 13.21% and 15.71%, separately (Fig. 2d). For PA and moderately differentiated acinar (MA), epithelial cells, including club, basal, and AT2 cells, were predominant cell types (Supplementary Fig. S8).

To explore the functions of macrophages in TME across histologic subtypes, we integrated two public scRNA-seq datasets^21,36^ to assess phenotypic differences of macrophages by multimodal intersection analysis (MIA)^37^. With the exception of anti-inflammatory macrophages that were prevalent across histologic subtypes, macrophage phenotypic heterogeneity was a key feature in LUAD (Fig. 2e). For example, tumor-associated macrophages (TAMs) were enriched in S subtype. The number of angiogenesis-associated macrophages was much larger in PA, MP and S subtypes. By contrast, phagocytosis-associated macrophages were depleted in PA and S subtypes. In addition, alveolar resident macrophages preferred to reside in normal tissues. These results revealed heterogeneity of LUAD histologic subtypes, and differential enrichment of cells in diverse histologic subtypes.

### Hypoxia in subtype progression

**Fig. 3:**
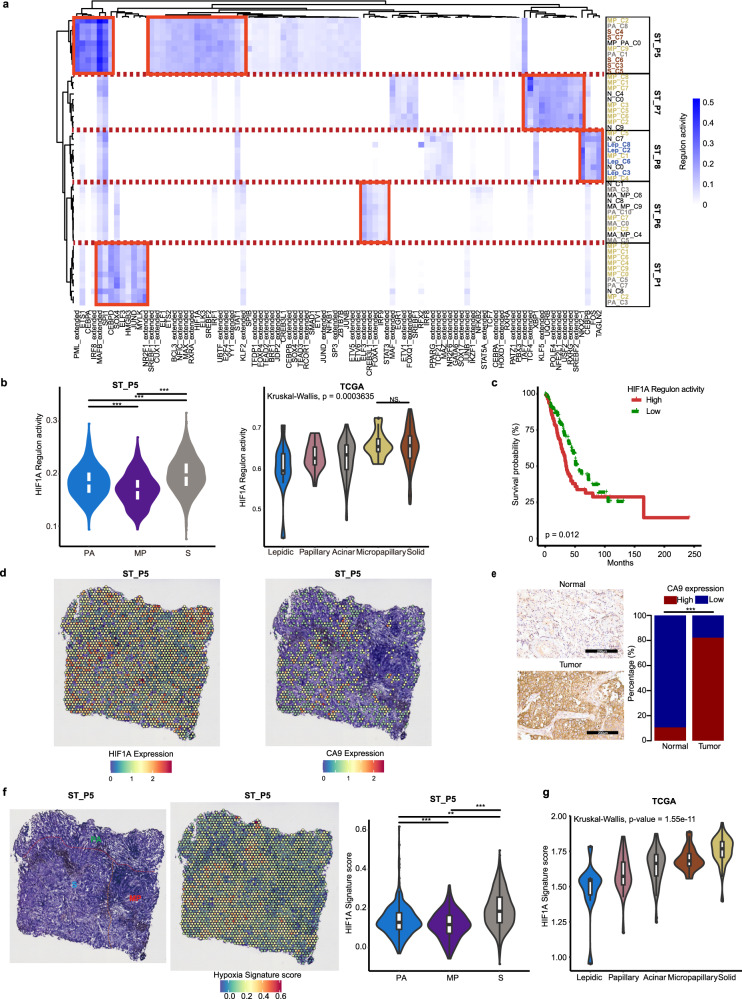
Transcriptional regulatory networks underlying subtype progression. **a** Identification and hierarchical clustering of regulons. The activity of regulons is indicated using color scale. The extended regulons represent regulons inferred by motif similarity. **b** HIF1A regulon activity in TCGA cohort (Lepidic, *n* = 11; Papillary, *n* = 28; Acinar, *n* = 71; Micropapillary, *n* = 23; Solid, *n* = 61). Kruskal–Wallis Rank sum test with Dunn’s multiple comparisons post hoc test. **c** Overall survival difference between high- and low-HIF1A regulon activity (Kaplan–Meier). Log-rank test. **d** The expression of hypoxia markers *HIF1A* and *CA9* examined by ST. **e** IHC staining of CA9 in tumors (*n* = 62) and normal tissues (*n* = 84). Chi-squared test. Scale bars, 200 μm. **f** Spatial plot of hypoxia score by signature genes, and statistical differences among different histologic subtypes. Kruskal–Wallis rank sum test with Dunn’s multiple comparisons post hoc test. **g** *HIF1A* signature score in TCGA cohort (Lepidic, *n* = 11; Papillary, *n* = 28; Acinar, *n* = 71; Micropapillary, *n* = 23; Solid, *n* = 61). Kruskal–Wallis rank sum test. *P < 0.05, **P < 0.01, ***P < 0.001.

To explore molecular mechanisms that shape the intrinsic differences of histologic subtypes, we performed regulatory network inference by SCENIC^38^, and identified regulons which consisted of transcription factors (TFs) and their downstream targets. *HMGB3*, a regulator of tumor proliferation^39^, and *UQCRB*, a subunit of the mitochondrial complex, were enriched in samples ST_P1 and ST_P7, respectively (Fig. 3a). Hypoxia-inducible factor 1α (*HIF1A*), which affects tumor initiation, invasion and metastasis^40^, had significantly higher regulon activity in S subtype compared with PA and MP subtypes (Fig. 3b). In The Cancer Genome Atlas (TCGA) cohort, *HIF1A* regulon activity increased from lepidic to high-grade subtypes. No significant difference was observed between S and MP subtypes, likely due to the histologic annotation of TCGA cohort was based on the predominant subtype, each sample in the TCGA cohort was annotated with only one histologic subtype. In addition, high *HIF1A* regulon activity correlated with worse overall survival (Fig. 3c). To further estimate varying hypoxia levels across histologic subtypes, we applied a hypoxia metagene signature to spatial spots^41^, including hypoxia markers *HIF1A* and *CA9* (Fig. 3d). Increased *CA9* expression in tumors was confirmed by IHC staining (Chi-squared test, *P* = 1.671e-17) (Fig. 3e). As expected, S subtype demonstrated a higher hypoxia signature score (Fig. 3f), and this observation was consistent with TCGA cohort (Fig. 3g).

### Progressive changes in cell signaling

**Fig. 4:**
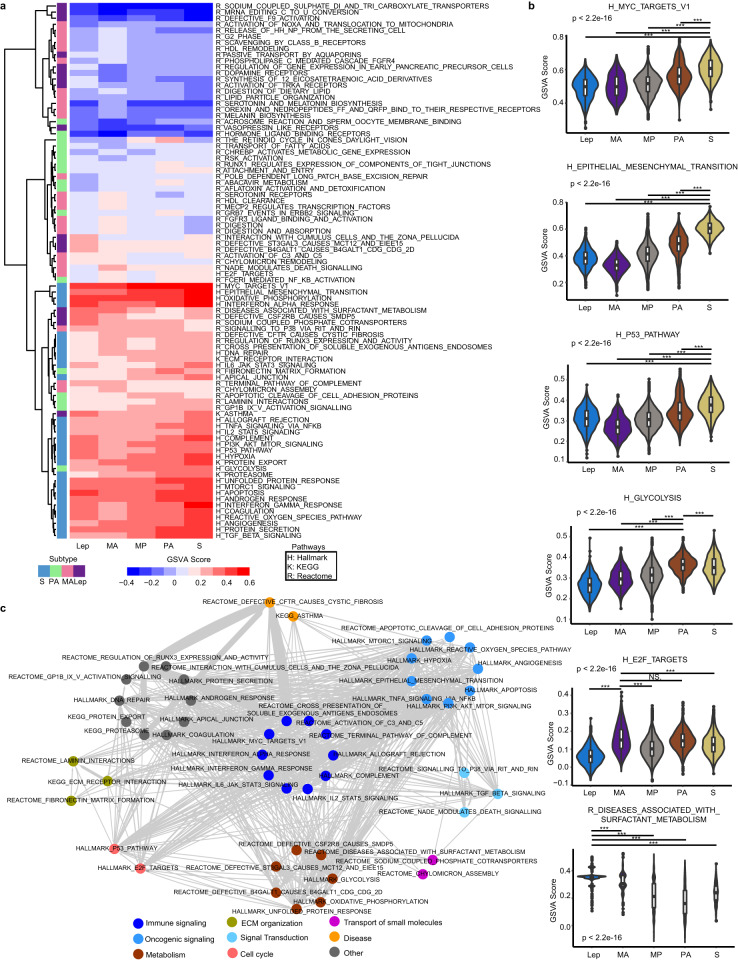
Characterization of cell signaling among histologic subtypes. **a** Enrichment analysis of hallmark gene sets (*n* = 50), KEGG pathways (*n* = 186) and Reactome gene sets (*n* = 1569). Each row represents a gene set, and each column represents an average GSVA score of the histologic subtype. GSVA score is indicated using color scale. **b** Representative violin plots for subtype-specific pathways. Kruskal–Wallis rank sum test with Dunn’s multiple comparisons post hoc test. **c** Intrinsic interactions between signaling pathways using Jaccard similarity. Nodes represent subtype-associated pathways, and edges represent Jaccard-weighed interactions between pathway pairs. *P < 0.05, **P < 0.01, ***P < 0.001.

To investigate the molecular consequences of regulatory networks and biological processes underlying subtype progression, we performed enrichment analysis of ~1800 gene sets from Molecular Signatures Database (MSigDB), including hallmark gene sets and canonical pathways. Subtype-specific pathways were identified (Fig. 4a). For example, MYC-regulated pathways, epithelial-mesenchymal transition, PI3K/AKT/mTOR signaling, P53 pathway, and hypoxia described above, were predominant biological processes in S subtype (Fig. 4b). Additionally, glycolysis and gluconeogenesis pathways were enriched in PA subtype, E2F-targeted genes were enriched in MA and PA subtypes, and surfactant metabolism is enriched in lepidic subtype (Fig. 4b).

We then assessed the intrinsic relationships between subtype-associated signaling pathways using Jaccard similarity (see Materials and methods). A biological network was constructed based on pathways (referred to as nodes) and Jaccard-weighed interactions between pathway pairs (edges) (Fig. 4c). Based on biological functions, these pathways had intrinsic interactions and were categorized into several classes, such as immune, cell cycle, oncogenic, metabolism, extracellular matrix (ECM) organization.

### Heterogeneity of dedifferentiation states across histologic subtypes

**Fig. 5:**
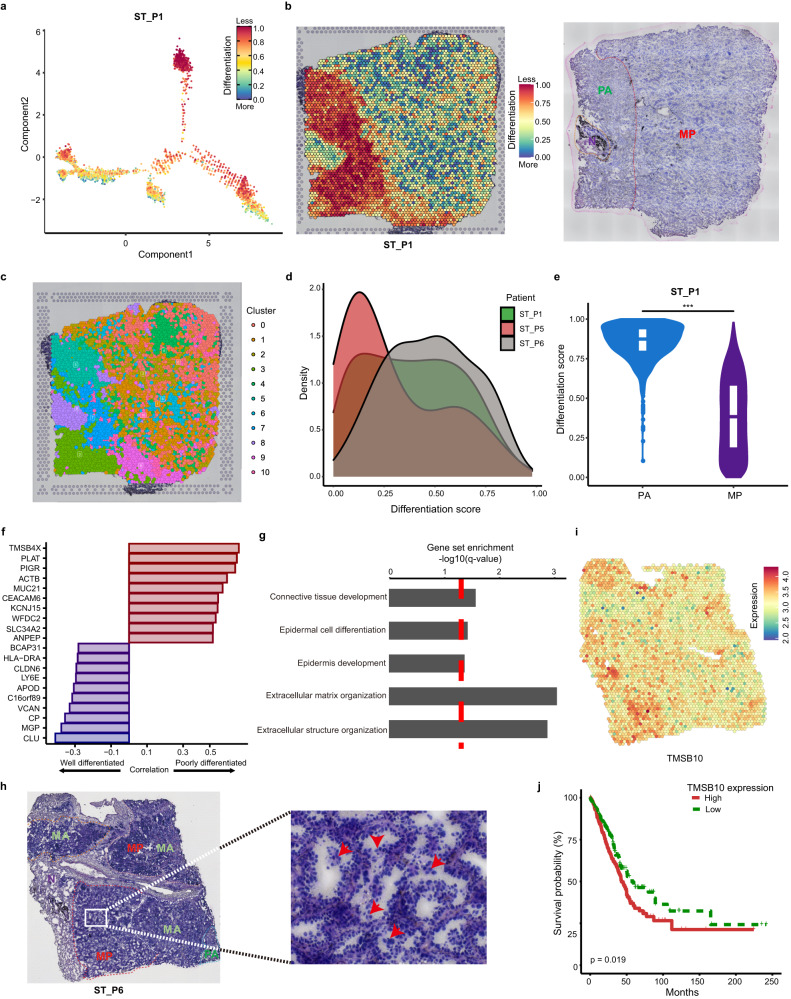
Heterogeneity of dedifferentiation states across histologic subtypes. **a** Differentiated trajectory of sample ST_P1 in a two-dimensional space inferred by Monocle2 and CytoTRACE. **b** Differentiation states of spatial spots and H&E staining of histologic subtypes. **c** Spatial distribution and clustering of ST_P1 ST spots. **d** The kernel density estimation of differentiation scores for MP subtype in our cohort. **e** Violin plots showing the comparison of differentiation scores between PA and MP subtypes. Wilcoxon Rank sum test. **f** Top 10 poorly differentiated genes and 10 well differentiated genes correlated with histologic subtypes. **g** Significantly enriched gene sets for differentiated genes. **h** MP subtype is featured by micropapillary tufts (red arrow) in ST_P6. **i** Spatial plot of micropapillary tufts-specific marker *TMSB10* expression in histologic section. **j** Overall survival difference between patients with high- and low-TMSB10 expression (Kaplan–Meier) in TCGA cohort. High expression (*n* = 250), low expression (*n* = 250). *P* value was calculated by log-rank test.

Dedifferentiation processes have been implicated in tumor progression^42^. The histologic subtypes of invasive LUAD are associated with prognosis, and high-grade subtypes that include MP, S and PA subtypes show a significantly increased risk of recurrence and metastasis^43^, and correlate with clinical response to checkpoint inhibitor therapies^44–46^. To gain insights into subtype-specific dynamically dedifferentiated changes, we performed differentiation analyses using Monocle2^47^ and CytoTRACE^48^, and applied principal component to visualize developmental trajectory (Fig. 5a; Supplementary Fig. S9a). ST_P1 comprised three histologic subtypes, MP, PA and normal (N) tissues (Fig. 5b). As expected, N region exhibited a more differentiated state, while a less differentiated state was observed in PA subtype, consistent with histologic annotations (Fig. 5b). In ST_P6, N region has also been demonstrated to be a more differentiated state, and MA, PA, MP subtypes displayed less differentiated states (Supplementary Figs. S10b, S11).

Interestingly, MP subtype, defined by the existence of small papillary tufts in alveolar space or connective tissues, showed a striking intra-subtype heterogeneity of differentiation states (Fig. 5b) and intermingled spatial clusters (Fig. 5c). Among the MP-specific clusters, Clusters 9 (Fig. 5c), localizing at the boundary of PA and enriching for AT2 cells, was less differentiated compared with other MP clusters (Supplementary Fig. S9b), suggesting a potentially transitional morphology in subtype progression. For the MP subtype, similar differentiation heterogeneity that spanned the whole differentiated states was observed in other samples (Fig. 5d). MP subtype displayed more differentiated potential than S subtype (Supplementary Fig. S10a). No significant difference was observed between MP and MA subtypes (Supplementary Fig. S10b), further indicating that MP subtype exhibited a moderately differentiated feature.

We next identified genes associated with dedifferentiation process in PA (less differentiated) versus MP (more differentiated) subtypes (Fig. 5e) by Pearson correlation. We found that dedifferentiation marker genes, such as *TMSB4X* and *PLAT*, were highly enriched in PA subtypes, whereas *MGP* and *CLU* were skewed towards well-differentiated subtypes (Fig. 5f; Supplementary Table S6). Meanwhile, development and differentiation pathways were significantly enriched as shown by gene ontology analyses (Fig. 5g; Supplementary Table S7).

In order to investigate more detailed molecular profiles of MP subtype, we dissected micropapillary tufts from spatial architecture (Fig. 5h), and found that high expression of *TMSB10* in these micropapillary tufts (Fig. 5i) was associated with significantly reduced overall survival (*P* = 0.019) (Fig. 5j). Previous studies also demonstrated *TMSB10* as a key regulator of tumor progression and metastasis^49^. Taken together, our findings revealed subtype-specific heterogeneity of dedifferentiation states in LUAD, especially MP subtype, which might explain the highly invasive feature of MP subtype.

### Subtype-specific immune landscape

**Fig. 6:**
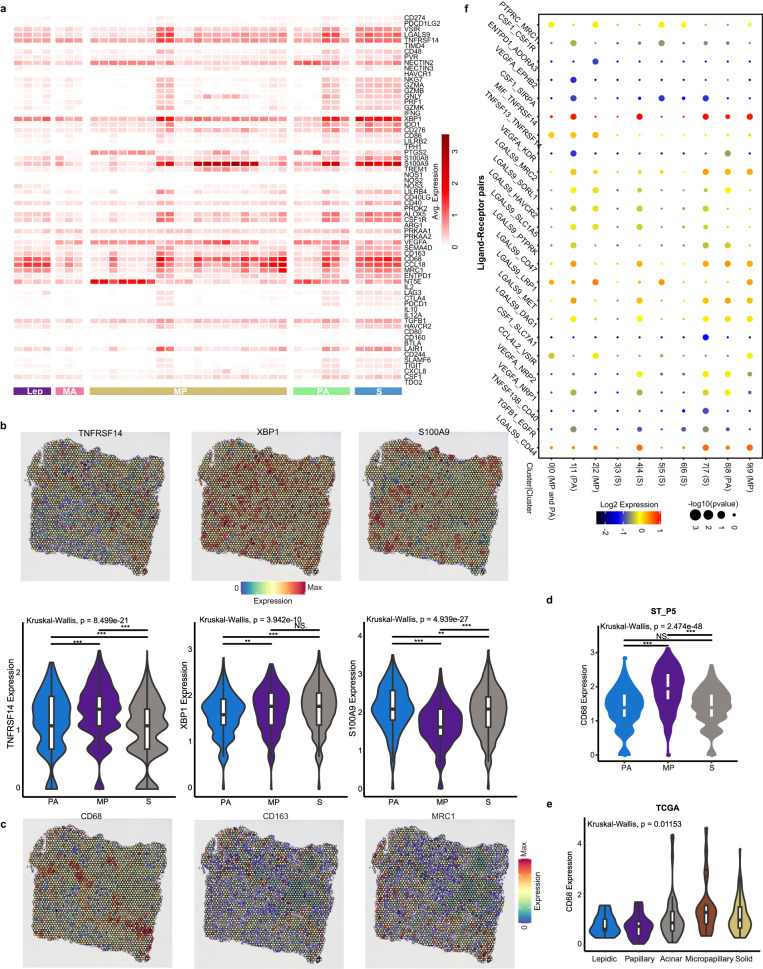
Immune profiles of histologic subtypes. **a** Heatmap showing expression of immunosuppressive genes in different histologic subtypes. **b** Spatial feature plots of representative genes, and corresponding expression differences among histologic subtypes. **c** Expression of M2-like macrophage marker genes (*CD68*, *CD163*, *MRC1*/*CD206*) in histologic section. **d**, **e** *CD68* expression in sample ST_P5 (**d**) and TCGA cohort (Lepidic, *n* = 11; Papillary, *n* = 28; Acinar, *n* = 71; Micropapillary, *n* = 23; Solid, *n* = 60) (**e**). Kruskal–Wallis rank sum test with Dunn’s multiple comparisons post hoc test. **f** Intra-cluster ligand-receptor pairs. Dot size represents statistical significance, and color for average expression of ligand and receptor.

To illustrate immune profiles of TME across LUAD subtypes, especially immunosuppression in TME, we explored the spatial distributions of immunosuppressive genes and co-signaling molecules that expressed in various immune cells (Fig. 6a), including dendritic cells (DCs), exhausted T cells, T regulatory (Treg) cells, myeloid-derived suppressor cells (MDSCs), TAMs, regulatory B (Breg) cells, and monocytes. High-grade (PA, MP and S) subtypes exhibited an immunosuppressive phenotype, especially S subtype. *TNFRSF14*, expressed by Tregs and antigen-presenting cells (APCs), delivered inhibitory signals by interacting with co-inhibitory factor *BTLA* (B and T lymphocyte attenuator) or *CD160* (upregulated in effector T cells^50^). DC-specific *XBP1* (blunts T cell anti-tumor immunity^51^), *S100A8* and *S100A9* (markers for MDSCs^52^), and *NT5E* (expressed by Tregs, converts ATP to adenosine that suppresses the activity of effector T cells and APCs)^53^, were highly expressed (Fig. 6a). *TNFRSF14* and *XBP1* were expressed prominently in MP subtype, while an opposite trend was observed for *S100A9* (Fig. 6b). A significant spatial correlation between *S100A9* and *NT5E* was observed in S subtype (Supplementary Table S8). Notably, M2-like macrophages (*CD68*^*+*^, *CD163*^*+*^, *MRC1*/*CD206*^*+*^), known as anti-inflammatory and pro-tumorigenic macrophages, were also abundant across histologic subtypes (Fig. 6a, c). The increase in expression of *CD68* in MP cluster (Supplementary Fig. S12a and Table S4) was validated in TCGA cohort (Fig. 6d, e).

To characterize cell-cell communication networks, we used CellPhoneDB^54^, allowing to integrate a repository of ligands, receptors, heteromeric complexes, and their interactions, to infer crosstalk pairs, especially spatial-dependent intra-cluster ligand-receptor complexes (Fig. 6f). Considering spatial distances between cells, we focused only on intra-cluster cell-cell interactions of each histologic subtype. *MIF*-*TNFRSF14*, a significantly enriched ligand-receptor pair, was highly expressed in MP, S and PA clusters. Aberrantly expressed macrophage migration inhibitory factor (*MIF*) (Supplementary Fig. S12b) was previously reported as a cytokine that regulates innate immunity^55^, overexpressed in various cancers and involved in M2-like macrophage polarization^56^. It promotes pancreatic ductal adenocarcinoma progression by the MIF-mir-301b-NR3C2 signaling axis^57^. We also identified a multifaceted ligand *LGALS9* (Supplementary Fig. S12c) binding to diverse receptors, including *HAVCR2* (*TIM-3*) which mediates immune suppression by T cells or macrophages^58^, immunotherapy checkpoint *CD47* that overexpressed in many tumor types^59^. Collectively, our data suggested that an immunosuppressive microenvironment, particularly an essential role of M2-like macrophages, may shape diverse histologic subtypes and facilitate LUAD progression

### Spatial profiles of macrophages across histologic subtypes by mIHC

**Fig. 7:**
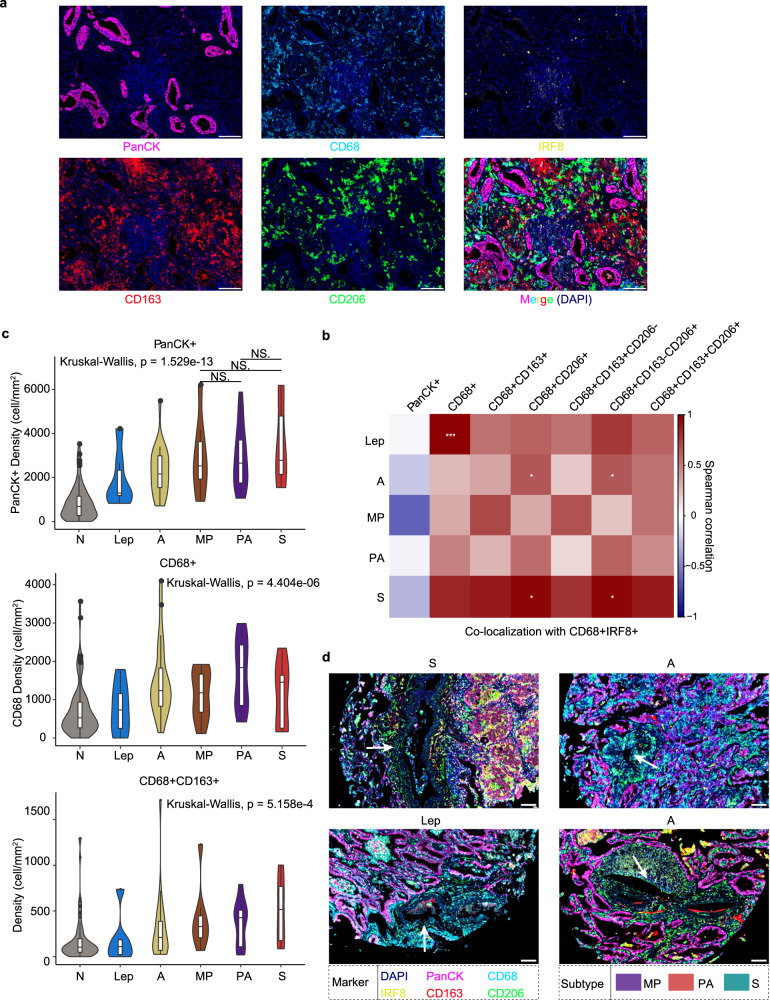
The landscape of macrophage subpopulations. **a** Representative illustration of a LUAD tissue stained by multiplex IHC. Scale bar, 100 μm. DAPI (blue), PanCK (pink), CD68 (cyan), IRF8 (yellow), CD163 (red), CD206 (green). **b** Co-localization of CD68^+^IRF8^+^ macrophages with other subpopulations based on spearman correlation of densities. **c** Densities of TAM subpopulations across histologic subtypes. Kruskal–Wallis rank sum test with Dunn’s multiple comparisons post hoc test. Normal (N), *n* = 87; lepidic (Lep), *n* = 6; acinar (A), *n* = 22; micropapillary (MP), *n* = 8; poorly differentiated acinar (PA), *n* = 17; solid (S), *n* = 7. **d** Enrichment of CD68^+^CD206^+^ macrophages in vessel regions. Scale bars, 100 μm. DAPI (blue), PanCK (pink), CD68 (cyan), IRF8 (yellow), CD163 (red), CD206 (green). *P < 0.05, **P < 0.01, ***P < 0.001.

As a predominant cell type (Fig. 2a), macrophages also exhibited immunosuppressive functions in TME (Fig. 6a). However, it remained unclear whether diverse TAM subpopulations contribute to lung adenocarcinoma progression, and how these macrophage subpopulations spatially distribute across different histologic subtypes. We then used an additional cohort comprising 92 tumors and 88 adjacent normal tissues to perform mIHC. The mIHC assay includes several macrophage markers located in the cell membrane, cytoplasm or nucleus (Fig. 7a): cytokeratin markers (Pan-CK) for tumor cells^60^; CD68, a highly expressed pan-macrophage marker which primarily localizes on lysosomes and endosomes, and also localizes on cell membranes^61^; interferon regulatory factor 8 (IRF8), also known as interferon consensus sequence-binding protein (ICSBP), an M1-like macrophage marker^62^; CD163 and CD206 that are upregulated in M2-like macrophages.

For each tumor tissue, stroma and tumor regions were separated by the expression of PanCK. The CD68^+^, CD68^+^CD163^+^, and CD68^+^CD163^+^CD206^–^ macrophages were abundant in both tumor and stroma regions. In contrast, CD68^+^IRF8^+^, CD68^+^CD163^+^CD206^+^ macrophages were less enriched in both tumor and stroma regions (Supplementary Fig. S13). Despite the low abundance of M1-like macrophages, we found significant co-localization of CD68^+^IRF8^+^ with CD68^+^CD206^+^ and CD68^+^CD163^–^CD206^+^ TAMs (Fig. 7b), reflecting a dynamic process of macrophage polarization in vivo, especially in acinar and solid subtypes. The density of tumor cells increased significantly from normal regions to solid subtype (Kruskal–Wallis, *P* = 1.529e−13), and was abundant in high-grade subtypes (MP, PA and S) (Fig. 7c). We also observed similar trends for CD68^+^CD163^+^ (Fig. 7c), CD68^+^CD163^+^CD206^–^ (Supplementary Fig. S14a) TAM subpopulations, but not in CD68^+^CD163^+^CD206^+^ (Supplementary Fig. S14b) subpopulation. In addition, the trend of CD68^+^ macrophages was consistent with subtype progression as well, but highest in PA subtype (Fig. 7c) as described previously^63^. The spatial distributions of TAMs across histologic subtypes were poorly understood. We then characterized the proximity of macrophage subpopulations to tumor cells using Euclidean distance based on pixel coordinates of the field of view. We found statistically significant differences in spatial distances between histologic subtypes in different TAM subpopulations, and the biological implications of this phenomenon remain elusive (Supplementary Fig. S15). Interestingly, we observed an enrichment of CD68^+^CD206^+^ macrophages in vessel regions in several samples (Fig. 7d; Supplementary Figs. S16–S19), suggesting that CD206-specific macrophages may promote tumor vascularization in a spatially dependent manner during tumor progression. In summary, our findings further supported the indispensable roles of TAM subpopulations in shaping diverse histologic subtypes during tumor progression.

## Discussion

**Fig. 8:**
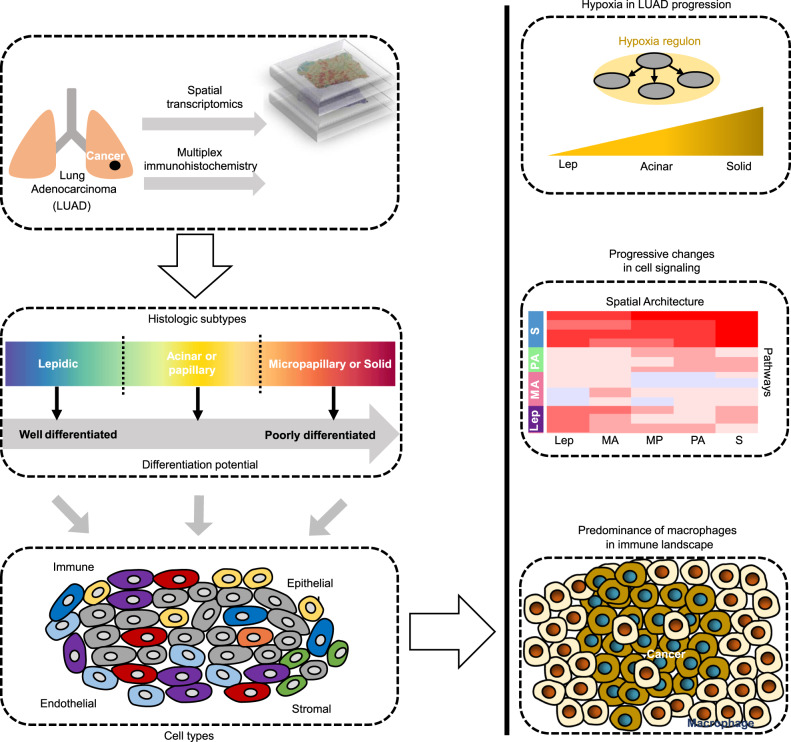
Schematic representation of transcriptional reprogramming and dynamic cell signaling underlying LUAD subtype progression and dedifferentiation states. Based on spatial transcriptomics (ST) and mIHC, we demonstrated cellular compositions, hypoxia regulon, cell signaling, and immune landscape during LUAD dedifferentiation and progression.

Intrinsic molecular features and extrinsic microenvironment determine tumorigenesis and metastasis^64^. Recent studies have revealed that driver mutations in LUAD were not associated with specific histologic subtypes, and oncogenic alterations didn’t drive subtype progression and spatial heterogeneity. Epigenetic and transcriptional reprogramming is a key determinant of histologic subtypes^63,65^. Meanwhile, substantial advances have been achieved by bulk RNA-seq, proteomics and single-cell profiles, while information on spatial localization of tumor cells, stromal cells and immune cells, as well as intercellular communications is lost during tissue dissociation. The lack of comprehensive spatial characterizations of TME remains an obstacle in improving therapeutic strategy and clinical prognosis^66^. Therefore, delineating histologic features at spatially resolved molecular resolution is critical to depict heterogeneity of histologic subtypes and corresponding molecular profiles in tumor progression. Here, we integrated spatial transcriptomics and mIHC in invasive lung adenocarcinoma to elucidate molecular mechanisms and microenvironment composition driving histologic subtype progression (Fig. 8).

Cellular composition and plasticity are fundamental to tissue morphogenesis and development. Most of lung adenocarcinoma are characterized by coexistence of more than two histologic subtypes in a tumor tissue. Therefore, it is important to comprehensively investigate cell lineage composition. We found that histologic subtypes demonstrated a high level of similarity for major cell types, but differential enrichment of cells in diverse histologic subtypes. However, it is important to note that cellular composition is inferred by a comprehensive cell atlas of the human lung comprising 58 cell subpopulations^23^. Other novel or rare cell types, especially intermediate cell states, may not be resolved or identified by current technologies. To overcome this limitation, we predicted differentiation state of each spatial spot using an unsupervised framework, and observed heterogeneity in dedifferentiation states among histologic subtypes, which were consistent with histologic morphologies. Interestingly, a recent study revealed a high-plasticity cell state (HPCS) during lung cancer progression using genetically engineered mouse models of human cancer. HPCS cells displayed robust potential for differentiation, as well as proliferation^24^. Importantly, the MP subtype exhibited substantially heterogeneous differentiation states, possibly explaining the occurrence of micropapillary tufts. Combining lineage-tracing techniques with spatial sequencing approaches could facilitate the construction of clonal trajectories and track cell differentiation dynamics during the progression of histologic subtypes^67^.

The most abundant cell type was macrophages in our dataset, possibly suggesting their essential roles in immune response, phagocytosis, angiogenesis, and lung homeostasis. TAMs regulate tumor immunity via immune checkpoint blockade, secretion of inhibitory cytokines, metabolic reprogramming, and recruitment of immunosuppressive cells^68^. Notably, we found different TAM subpopulations in different histologic subtypes, indicating the indispensable roles of TAM subpopulations in remodeling tumor environment and mediating immunosuppression to promote tumor progression. Although precise cellular interactions between tumor cells and TAMs are still not fully understood, our results help to improve our understanding of cellular relationships within TME, which may promote the development of therapeutic approaches that target spatial architectures. As new single-cell resolution spatial transcriptomics will become available, we will better elucidate cell-cell interactions and cell state transitions in spatial architecture.

In addition, there were a large number of T cells in TME. T cells, including naïve T cells, effector T cells (T helper cells, cytotoxic CD8^+^ T lymphocytes) and memory T cells (central memory T cells, effector memory T cells), mediate adaptive immune response. Macrophages act as APCs to activate T cells through MHC/TCR interactions and costimulatory signaling. The T helper cells secret cytokines, recruit innate immune cells and activate antitumor immunity. The cytotoxic CD8^+^ T lymphocytes (CTLs) execute effector function and promote cell death by perforin-granzyme and Fas/FasL pathways^69^. Dysfunctional or exhausted T cells in TME are characterized by overexpression of inhibitory checkpoint molecules, such as PD-1, TIM-3, LAG3, CTLA4 and TIGIT^70^. Recently, Paolo D. A. Vignali et al. revealed that exhausted T cells could limit antitumor immunity by generating immunosuppressive adenosine through hypoxia-induced CD39^71^. Meanwhile, Treg can inhibit antitumor immune response by suppressing APCs or producing immunosuppressive cytokines to downregulate effector T cells^72^. Reversing T cell dysfunction has been emerging as a promising therapeutic approach against tumorigenesis. In addition, immune checkpoint therapy (PD1/PD-L1 and CTLA4), adoptive T cell immunotherapy also have demonstrated great achievements in clinical efficacy^73^.

Molecular mechanisms underlying lung adenocarcinoma progression remain less well-characterized. Annotation of tumor samples only by histologic features lacks the information on intratumor heterogeneity or histologic subtypes. Gene regulatory networks, including transcription factors and target genes, determine transcriptional state of a cell. We found that differentiation- and proliferation-related regulons, such as *HMGB3*, modulated cellular plasticity and reprogramming, and potential cell state transitions. Moreover, we observed significant hypoxia during subtype progression. As a common phenomenon in tumors, hypoxia stimulates dissemination of tumor cells to other organs by altering cell–cell interaction and ECM, promotes angiogenesis, M2 polarization of TAMs, neutrophil infiltrating, and induces immunosuppression by recruiting MDSCs and Tregs^74^. Intriguingly, airway stem cells can sense hypoxia, thus differentiating into epithelial neuroendocrine cells that secret peptides and amines, which mitigate hypoxic injury^75^. Further investigations are needed to elucidate the associations between hypoxia and cellular dedifferentiation.

In summary, our results delineated dynamic biological processes and dedifferentiation states during subtype progression. Spatially resolved molecular profiles provide a detailed and unbiased map of positional context of cancer cells and TME during tumor progression, and enable us to directly scrutinize molecular features and heterogeneity of cell dedifferentiation states underlying subtype progression, thereby yielding potentially novel insights for therapeutic options.

## Materials and methods

### Human specimens

Five treatment-naïve patients with early-stage LUAD undergoing radical lobectomy at the Department of Thoracic Surgery II, Peking University Cancer Hospital & Institute were prospectively recruited for ST profiling from June 2020 to October 2020 (Supplementary Table S1). Tissue slices of ~3-mm thick were cut immediately after operation from each patient and then divided into several 6.5 × 6.5 mm fragments. The small tumor fragments were embedded in optimal cutting temperature compound (OCT, #4583, Sakura, Torrance, USA) and frozen on dry ice. After frozen, all tumor fragments were stored at –80 °C and used for ST within three months after surgery. Two experienced thoracic pathologists reviewed every slide to identify the appropriate fragments for ST, and annotated tumor slide for distinct histologic subtypes, including lepidic (Lep), papillary (P), acinar (A), micropapillary (MP) and solid (S) subtypes. The pathological diagnosis of individual sections was performed according to the 2015 WHO classification of LUAD and the new grading system proposed by International Association for the Study of Lung Cancer pathology committee^9,76^.

This study was conducted according to the Declaration of Helsinki (as revised by 2013) and approved by the Ethics Committee of Peking University Cancer Hospital & Institute (Institutional Review Board No. 2019KT59). All patients provided written informed consent before enrollment in this study.

### Slide preparation, staining, and imaging

Spatial transcriptomics arrays include four identical 6.5 mm × 6.5 mm capture areas, and each with 4992 spatial-barcoded spots (10× Genomics). Every spot has a diameter of 55 μm, and surrounding with six spots with a center-to-center distance of 100 μm. Tissues were cryosectioned, cut at 10-μm thickness, and then mounted onto spatial slides. Using the Thermocycler Adaptor with the active surface facing up, sections were incubated for 1 min at 37 °C. The slides were then fixed by methyl alcohol for 30 min at –20 °C, followed by H&E staining (Eosin, Dako CS701, Hematoxylin Dako S3309, bluing buffer CS702) and brightfield imaging with a Leica DMI8 whole-slide scanner at 10× resolution.

### Permeabilization and reverse transcription

Visium spatial gene expression slide and Reagent Kit (10× Genomics, PN-1000184) was used to detect spatial gene expression. Slides were inserted into slide cassettes that created leakproof wells for permeabilization with 70 μL enzyme, and incubated at 37 °C for 18 min. Each well was washed with 100 μL SSC, and added 75 μL reverse transcription (RT) Master Mix for cDNA synthesis.

### cDNA library preparation and sequencing

Removing RT Master Mix from the wells after the end of first-strand synthesis. Wells were incubated for 5 min at room temperature after adding 75 μL 0.08 M KOH, then washed with 100 μL EB buffer. Second-strand synthesis was performed by adding 75 μL Second Strand Mix to each well.

Visium spatial libraries were constructed using Visium spatial Library construction kit (10× Genomics, PN-1000184) according to the manufacturer’s instructions. The libraries were sequenced on Illumina Novaseq 6000 platform with pair-end 150 bp (PE150) strategy (performed by CapitalBio Technology, Beijing).

### ST data processing

Raw sequencing data were processed using Space Ranger pipelines (V1.1.0, https://support.10xgenomics.com/spatial-gene-expression/software/downloads/latest), including tissue detection, fiducial detection, read alignment, barcode and UMI counting against GRCh38 genome assembly and corresponding GENCODE annotation file (V32). Feature-spot matrices were generated based on spatial barcodes, and then analyzed with the Seurat R package (V3.2)^29^.

To normalize sequencing depth variance across spatial spots, especially for technical artifacts and tissue anatomy, we used SCTransform function based on regularized negative binomial regression to normalize molecular count data, and detect high-variance features. Dimensionality reduction was performed with principal component analysis (PCA), then followed by a shared SNN construction based on Jaccard index between spots with the first 30 dimensions. Cluster determination was performed using the FindClusters function at resolution 0.6 by a SNN modularity optimization. The top 30 PCA dimensions were used for UMAP dimensional reduction. Subsequently, clusters in UMAP space were visualized by DimPlot and SpatialDimPlot functions. Spatially variable features that correlate with spatial subtypes were identified by FindSpatiallyVariables function with markvariogram method. To identify differentially expressed genes for each cluster, we used FindAllMarkers function in Seurat with default parameters, and genes with logFC > 0.25 and adjusted *P* value < 0.05 were considered as significant different.

### Prediction of cell type composition using integration method

Each spatial spot with a diameter of 55 μm encompasses multiple cells. To infer the underlying composition and proportions of cellular subtypes in every spot, we employed two approaches to depict each spatial voxel. Firstly, using a molecular cell atlas of human lung that included 58 cell types as a reference scRNA-seq dataset^23^, we identified a set of anchors between reference and our ST data that subsequently were transferred to query object via FindTransferAnchors and TransferData functions in Seurat^77^. Secondly, we performed enrichment analysis using cumulative hypergeometric distribution by investigating the overlap between ST DEGs and cell type-specific marker genes. R packages ggplot2 (V3.3.0)^78^, export (V0.3.0) (https://github.com/tomwenseleers/export), and Patchwork (V1.0.1) (https://cran.r-project.org/web/packages/patchwork/index.html) were used for visualization.

In order to evaluate cellular composition across LUAD subtypes, we calculated hamming distance between each pair of clusters using R package e1071 (V1.7-3) (https://cran.r-project.org/web/packages/e1071/). Heatmap was drawn by R package pheatmap (V1.0.12) (https://cran.r-project.org/web/packages/pheatmap/index.html).

### Spatial correlation analysis

To assess cell type co-occurrence in a spot, we calculated Pearson correlations between pairwise cell types using all spatial spots, and used Holm adjustment for multiple tests via corr.test function in R package psych (V1.9.12.31) (https://personality-project.org/r/psych). Significant correlations were defined as follows: coefficient ≥ 0.3 and adjusted *P* value < 0.05.

Pairwise genes correlation was performed by nearest neighbor analysis. For each spot, normalized gene expression across the central spot and six surrounding nearest neighbors was calculated. We then calculated Pearson correlations as described above.

### Dedifferentiation trajectory inference

To characterize dedifferentiation transitions between histologic subtypes, we applied Monocle (V2.18.0) algorithm^47^ with spatial DEGs. Firstly, Size factors and dispersions were estimated for a new CellDateSet object that was created with count matrices. Genes expressed in less than 10 spots were filtered. Then dimension reduction using DDRTree algorithm, and ordering along pseudotime were performed. Secondly, we leveraged CytoTRACE^48^, based on decreased transcriptional diversity during differentiation, to infer dedifferentiation trajectories at spot resolution.

### Gene Ontology (GO) enrichment analysis

For genes associated with differentiation, R package clusterProfiler (V3.16.1)^79^ was used to perform GO enrichment analysis using Gene Ontology gene sets^80^. *P* value was adjusted for multiple comparisons by Benjamini-Hochberg correction. Significant ontologies were determined by a *q*-value cutoff of 0.05.

### TCGA data analysis

The normalized bulk RNA-seq data (*n* = 515) and corresponding predominantly histologic subtypes of LUAD were downloaded from TCGA LUAD cohort^81^. Only primary tumor samples were considered for downstream analysis. To characterize state transitions between histologic subtypes, patients without pathology information were excluded.

### Regulon inference

Gene regulatory networks (regulons) were identified using SCENIC (V 1.2.2)^38^ based on count matrices. Genes detected in < 1% of all spots were excluded. Co-expression network for potential TF targets was first calculated using random forest algorithm implemented in GENIE3. TF binding motifs enriched on transcription start site for hg38 reference genome were downloaded from cisTarget (https://resources.aertslab.org/cistarget/). We performed TF motif enrichment analysis and identified target genes of each TF module using RcisTarget. Next, we used AUCell to score regulon activity using area under the recovery curve across gene expression rankings for each spot, and generating a binary regulon activity matrix. Differentially activated regulons across histologic subtypes were identified by Wilcoxon Rank Sum test.

### Gene set variation analysis (GSVA)

A non-parametric and unsupervised method GSVA (V1.32.0)^82^ was used to estimate variation of gene set enrichment score or pathway activity. Hallmark gene sets (50 gene sets), and canonical pathways from C2 curated gene sets that comprised KEGG (186 gene sets) and Reactome (1569 gene sets), were obtained from MSigDB (V7.3). Single sample GSEA (ssgsea) method in gsva function was used to assess a gene set enrichment score for each spot or sample.

For further validation of regulon activity in an independent cohort, the enrichment score of each regulatory network (transcription factors and target genes) was evaluated across different histologic samples.

To unveil the underlying interactions among subtype-specific pathways, we quantified the degree of pathway similarity between each pathway pair using Jaccard index, which was defined as the ratio of the interaction size of two pathways over their union size. Next, a biological network was constructed: each pathway was defined as a node, and interaction weighed by Jaccard index between two pathways as an edge, and then visualized by Cytoscape (V3.6.0)^83^.

### Spatial ligand-receptor interaction analysis

Spatial intra-cluster ligand-receptor interaction pairs of histologic subtypes were inferred using CellPhoneDB (V2.1.4)^54^, a repository of ligands, receptors, heteromeric complexes, and their interactions. Metadata and count matrix files were used as input data, and the threshold (percentage of spots expressing a gene) was set to 0.4. Through 1000 randomized permutations of spatial cluster labels, we generated a null distribution on the basis of the average expression of ligand-receptor pair in the interacting populations. *P* value was calculated using the proportion of means that exceeded the actual mean, and ranked based on its significance.

### mIHC staining

LUAD tissue microarray (TMA), including formalin-fixed paraffin-embedded 92 tumor tissues and 88 normal tissues, was purchased from National Human Genetic Resources Sharing Service Platform (2005DKA21300). To identify the spatial distribution and heterogeneity of TAMs (M1-like and M2-like) in LUAD, we used PANO 7-plex IHC kit (cat 0004100100, Panovue, Beijing, China) to perform mIHC staining (mIHC) according to the manufacturer’s protocol. Antibodies included anti-CD68 (CST76437), anti-CD163 (CST93498), anti-CD206 (ab64693), anti-IRF8/ICSBP (sc-365042), anti-PAN-CK (CST4545S), anti-DAPI (SIGMA-ALDRICH, D9542). Briefly, The TMA was dewaxed and rehydrated. Then antigen retrieval by microwave was performed as follows: placing slide in 100 mL of antigen retrieval solution (Citric acid solution), boiling, cooling down, and washing with distilled water, then transferring slide to 1× TBST containing slide jar (TBS (pH 7.4) plus 0.1% Tween 20). Next, TMA was covered with blocking solution and incubated for 10 min. Primary antibody was added and incubated at room temperature, followed by horseradish peroxidase-conjugated secondary antibody and tyramide signal amplification. Sequential antibodies were stained by repeating the procedures as described above. After labeling all antigens, nuclei were stained with DAPI (SIGMA-ALDRICH, D9542). Finally, mIHC slides were scanned using Mantra System (PerkinElmer, Waltham, Massachusetts, US), PanoVIEW VS200 slide scanner (Panovue, Beijing, China) with Olympus 20× lens, and Polaris System (PerkinElmer, Waltham, Massachusetts, US).

### mIHC image quantifications

Quantifications and downstream spatial analyses were performed by inForm image analysis software (V2.4, PerkinElmer, Waltham, Massachusetts, US) and QuPath image analysis software (V0.2.0, the Queen’s University of Belfast, Northern Ireland, UK)^84^ on PanoATLAS workstation, including:

#### Tissue compartment types

Based on tissue morphologies, we separated each tumor tissue slide into tumor and stroma categories using feature recognition algorithms, and analyzed corresponding area for each category.

#### Cell count and density

Cells were counted by inform software for each cell type, and density of cells was calculated by cell count and normalized by pixel area (cell/mm^2^) in each slide.

#### Cells distance and co-localization

The Euclidean distance was adopted to describe cells’ spatial relationship based on pixel coordinates of the field of view. Cell type X and Y co-localization analysis was measured by the spearman correlation between X and Y densities across all slides.

### Survival analyses

We applied survival (V3.2-10) and survminer (V0.4.6) packages in R statistical software to assess the association between gene expression and overall survival time. We used log-rank test to calculate groups’ difference, and Kaplan–Meier method implemented in ggsurvplot function to plot survival curves.

### Statistical analyses

The Kruskal–Wallis rank sum test was applied to calculate statistical difference in independent samples from multiple groups using R package stats (V3.6.0), and Wilcoxon Rank Sum test for two-group comparison. For multiple comparisons, Dunn post hoc test was performed with R package FSA (V0.9.1) (https://github.com/droglenc/FSA). *P* value was adjusted for multiple hypothesis testing by Bonferroni, Benjamini-Hochberg or holm method. A chi-squared test was performed to compare the statistical difference in CA9 IHC between tumors and normal tissues.

### Supplementary information

Supplementary Figures

Supplementary Tables

## Data Availability

The raw spatial transcriptomics data can be obtained from the Genome Sequence Archive in the National Genomics Data Center^85^, China National Center for Bioinformation/Beijing Institute of Genomics, Chinese Academy of Sciences, under accession number HRA001238. The source codes used in this study can be found at https://github.com/minqing1/ST_LUAD.
