# Supplementary material for: Spatial transcriptomics delineates molecular features and cellular plasticity in lung adenocarcinoma progression
Source: Cell Discov. 2023 Sep 19;9:96. doi: 10.1038/s41421-023-00591-7 (PMC10507052; doi:10.1038/s41421-023-00591-7)
Supplement: Supplementary file 1 — Supplementary Figures [file 41421_2023_591_MOESM1_ESM.pdf]

Supplementary Figures

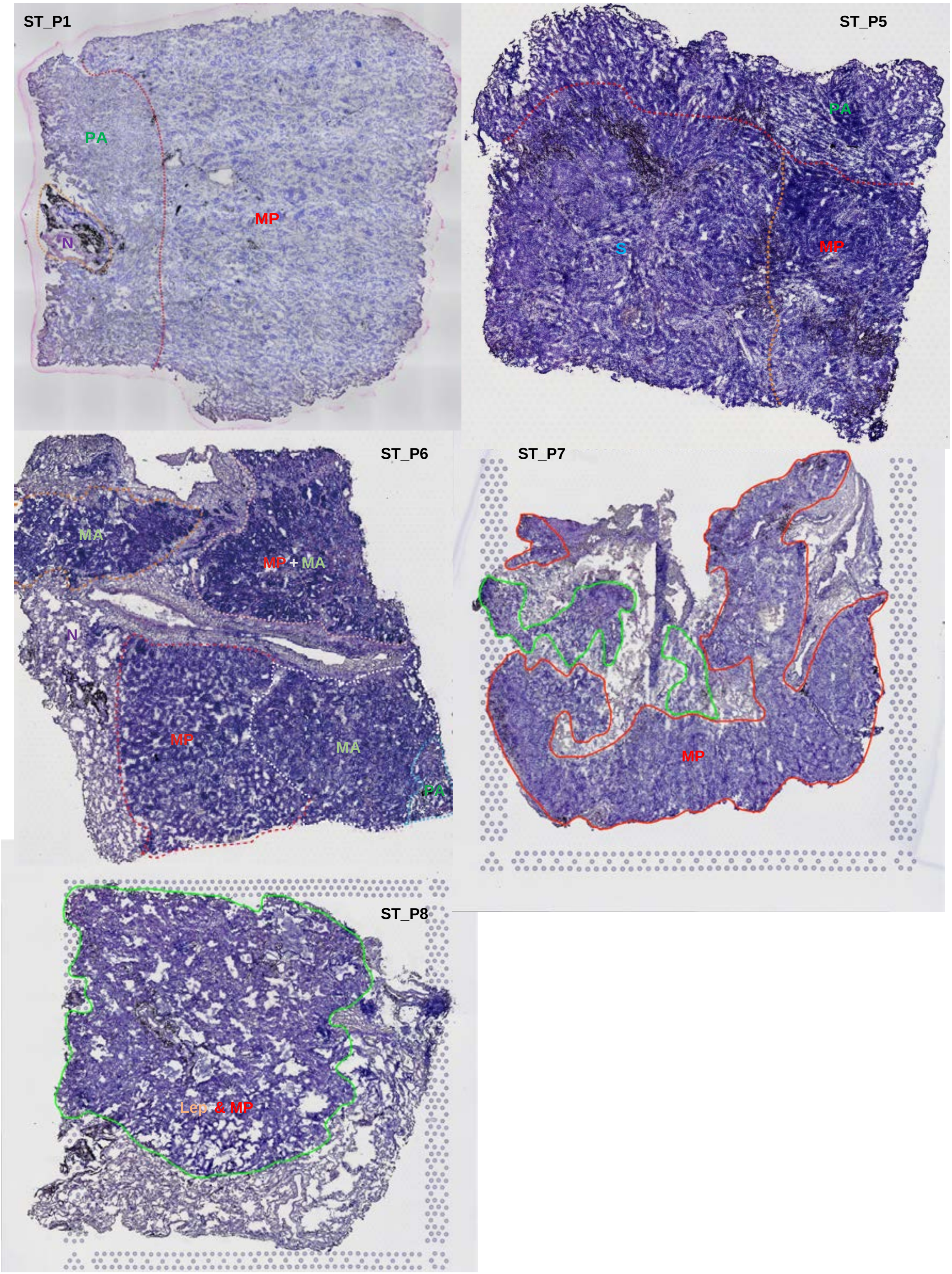

Supplementary Fig. S1 The annotation of histologic subtypes for all five tissue samples.

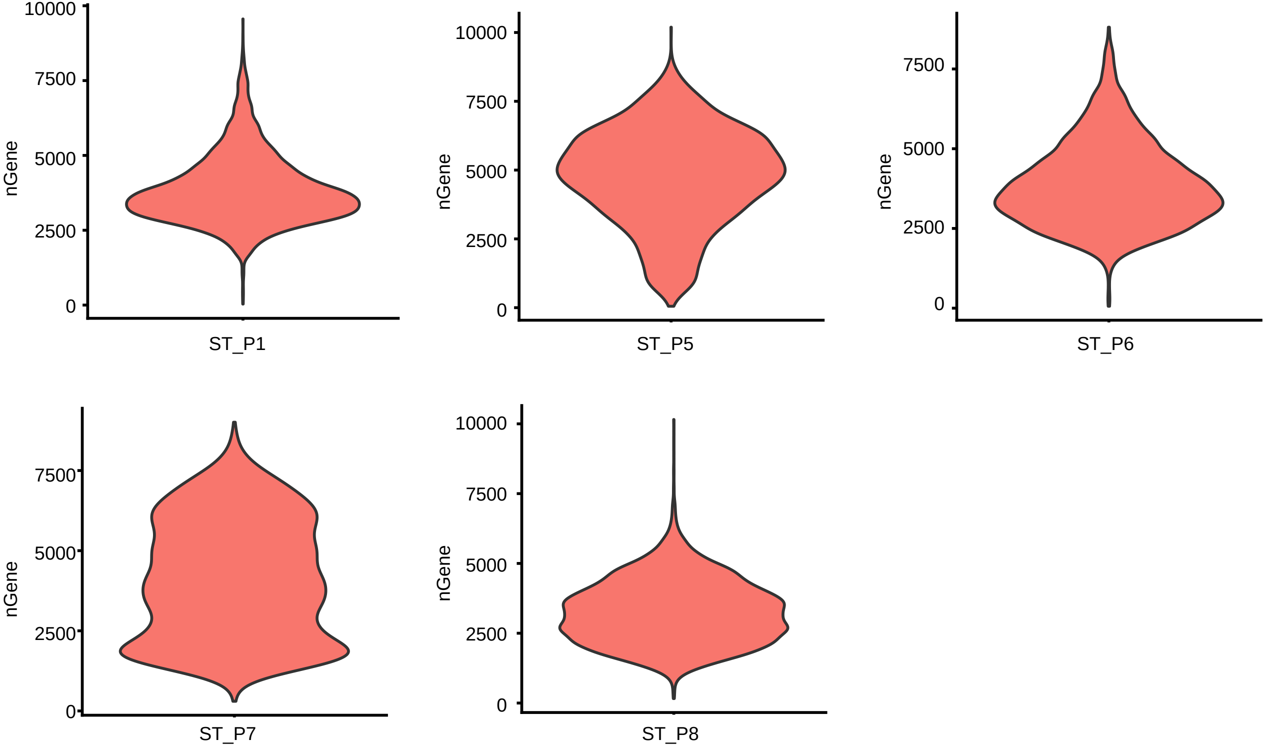

Supplementary Fig. S2 The number of genes detected in each spot among five ST patients.

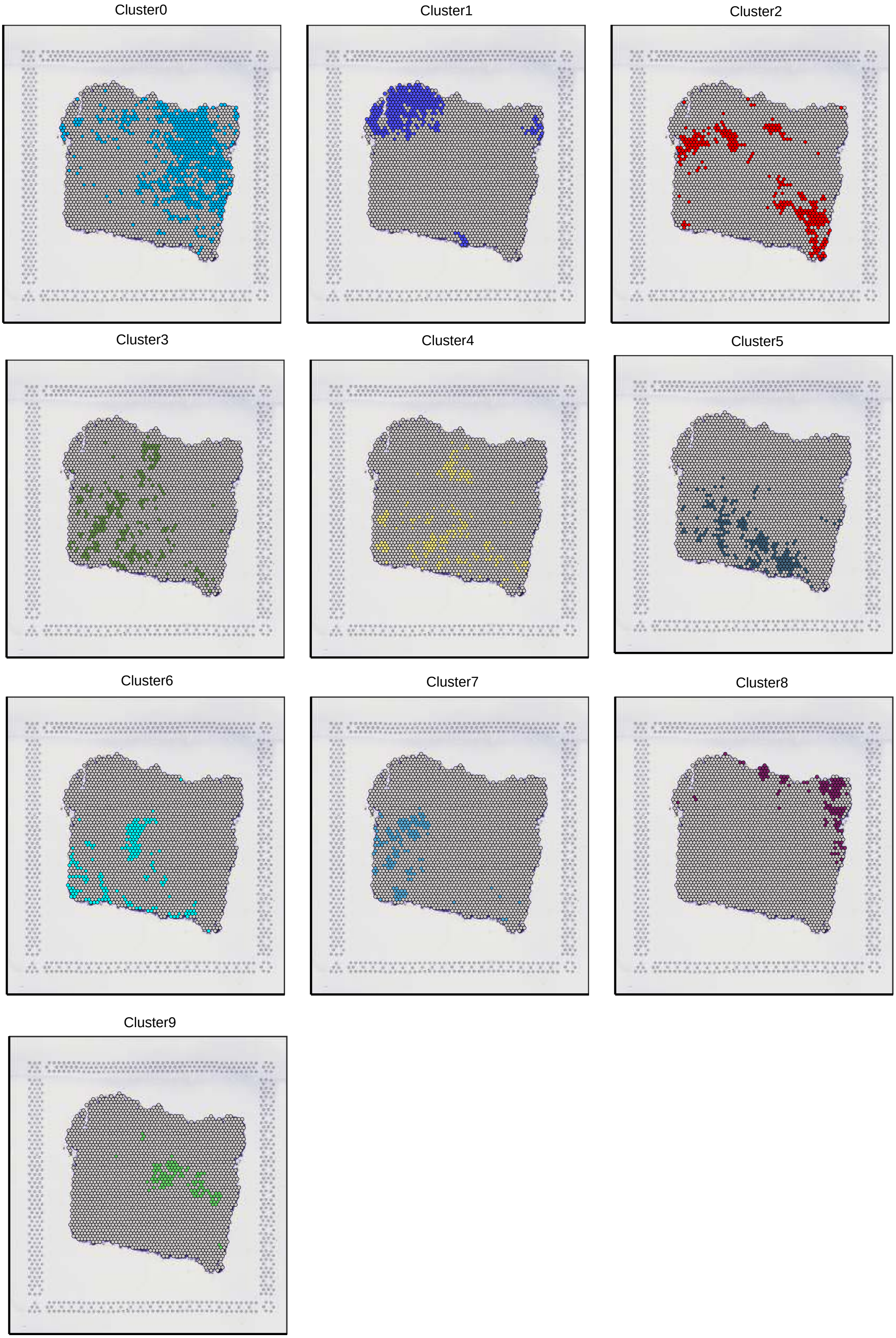

Supplementary Fig. S3 Spatial localization of each cluster for ST_P5.

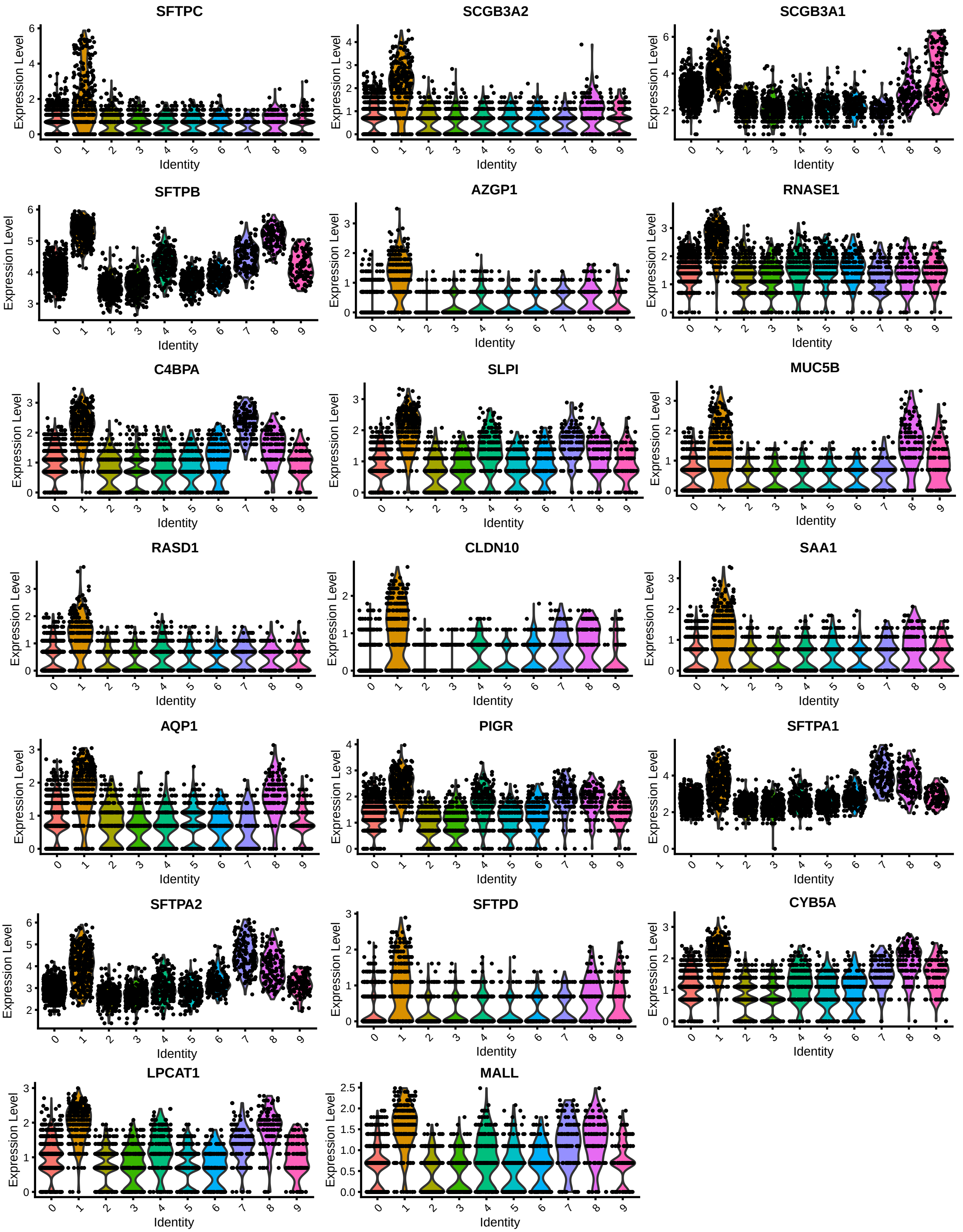

Supplementary Fig. S4 Top 20 differentially expressed genes of ST_P5 cluster 1.

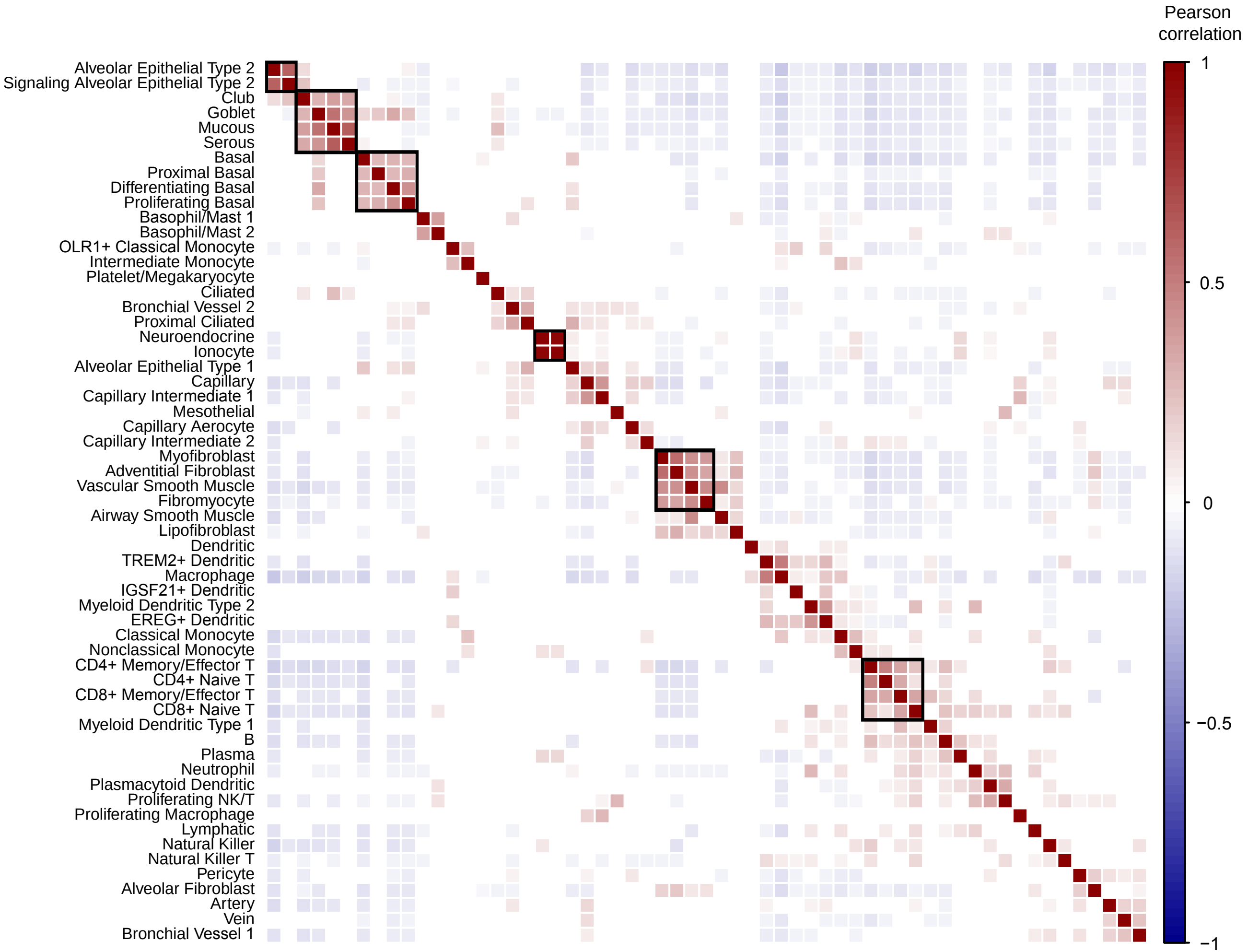

Supplementary Fig. S5 Spatial Pearson correlation of pairwise cell types. White represented non-significant correlations. Black boxes showed positively correlated cell types.

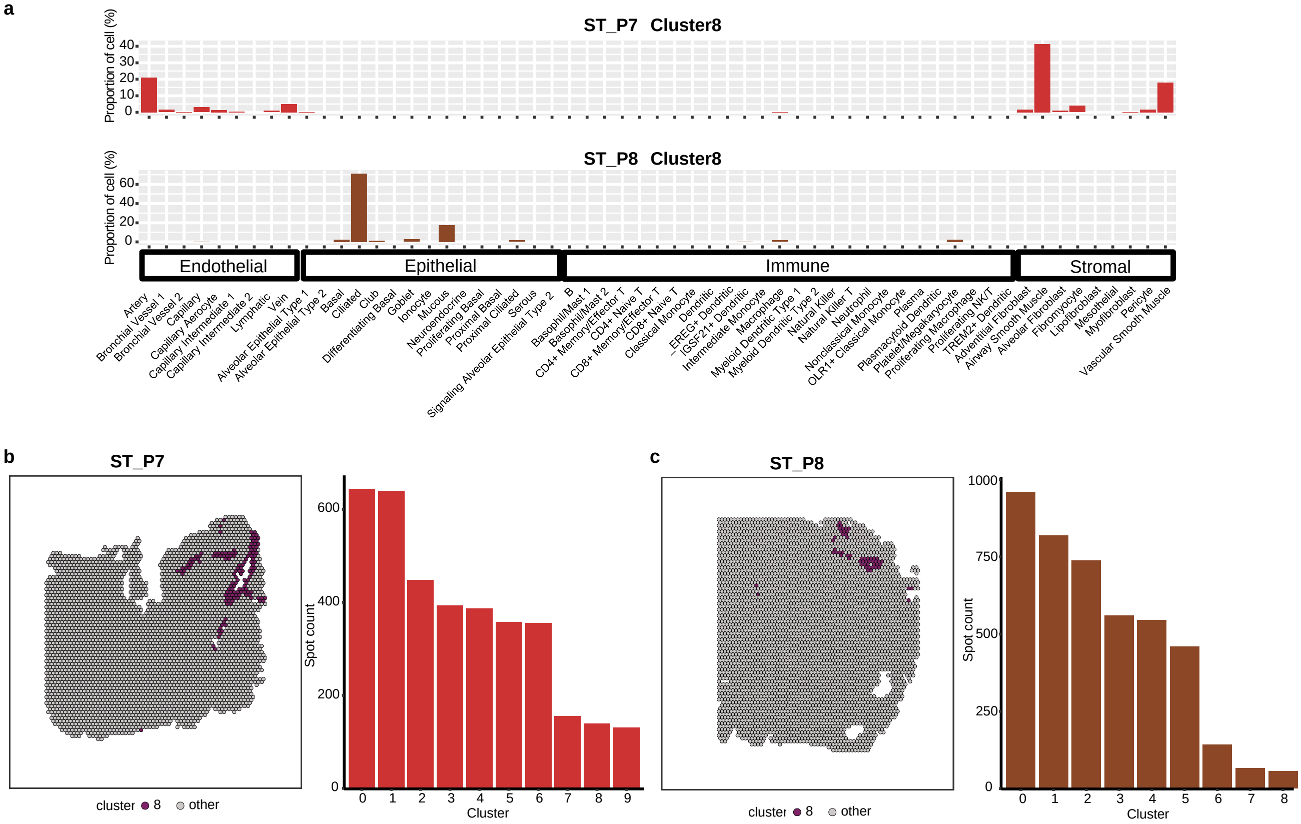

Supplementary Fig. S6 Cellular composition and spatial localization of two abnormal clusters. a The average proportion of each cell type in ST_P7 cluster 8 and ST_P8 cluster 8. Spatial localization of **b** ST_P7 cluster 8 and **c** ST_P8 cluster 8 in histologic section. Bar plots showing the number of spots covered by each cluster.

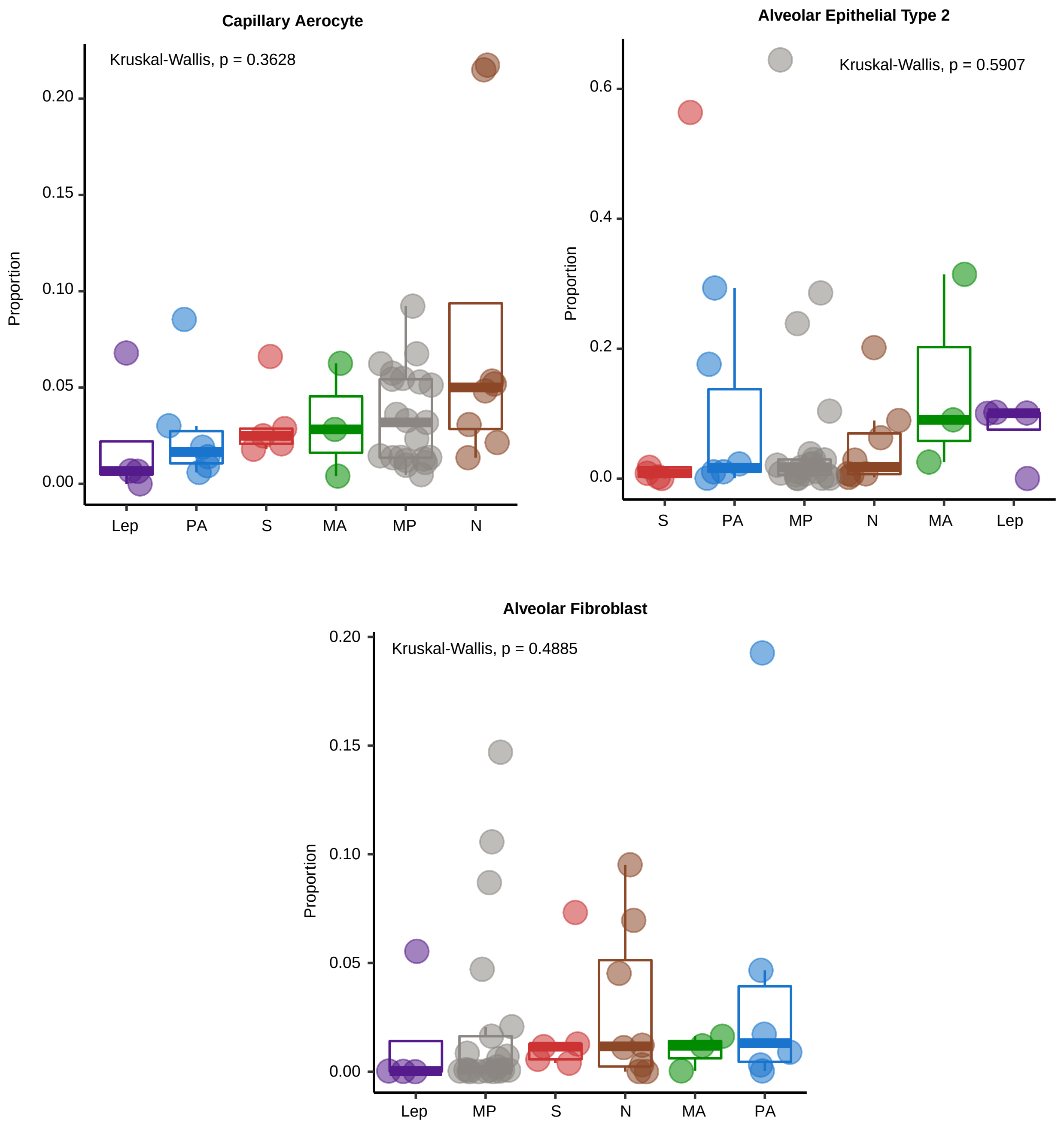

Supplementary Fig. S7 Cellular proportions of diverse cell types. Proportion differences of capillary aerocyte, alveolar epithelial type 2 and alveolar fibroblast cells among histologic subtypes. P values were calculated by Kruskal-Wallis rank sum test.

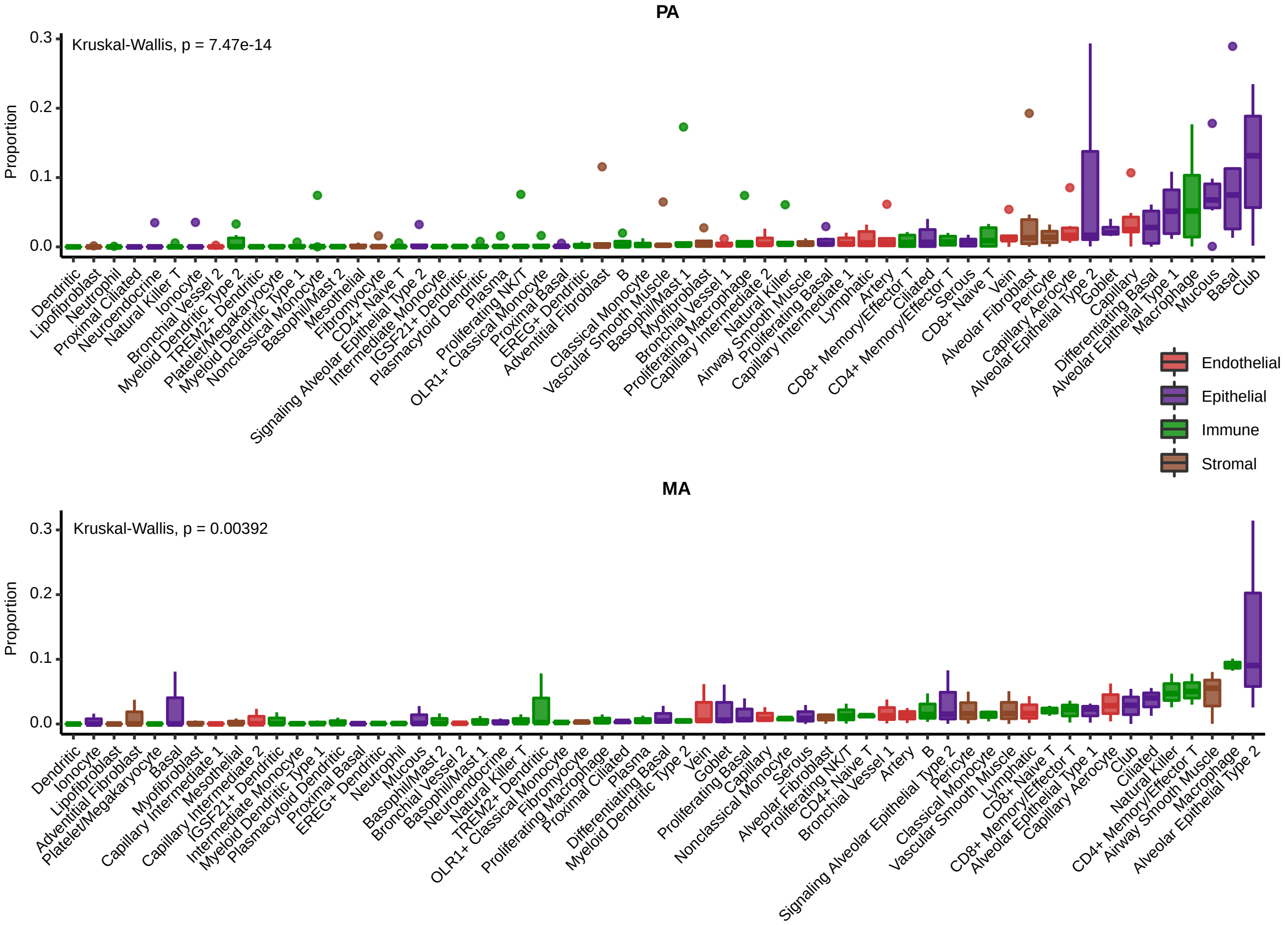

Supplementary Fig. S8 Cellular proportions of PA and MA subtypes. Proportion differences of PA and MA subtypes. P values were calculated by Kruskal-Wallis rank sum test.

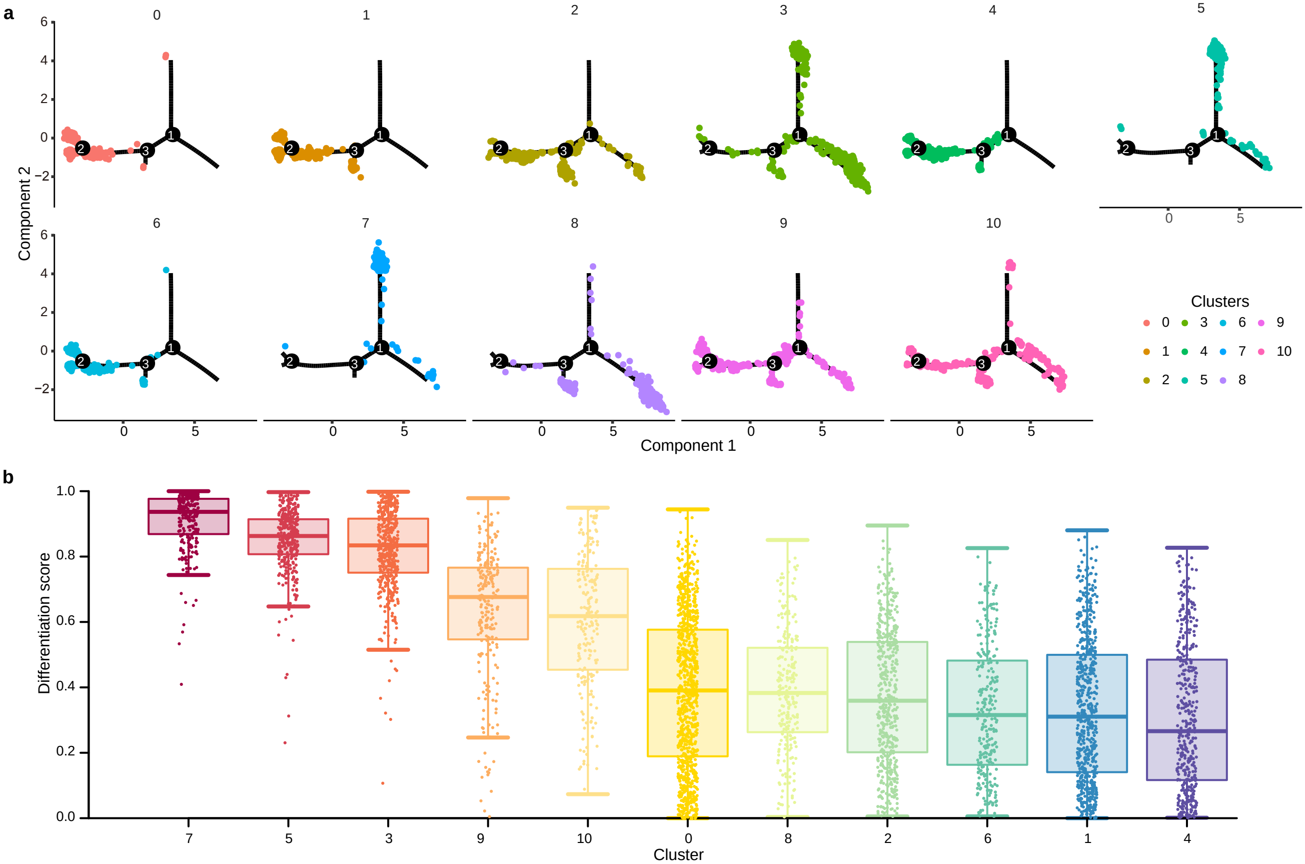

Supplementary Fig. S9 Differentiation trajectories and states of ST_P1. a Differentiated trajectory of each cluster for patient ST_P1 in a two-dimensional space inferred by Monocle2 and CytoTRACE. **b** Boxplots showing the distribution and median of differentiation scores per cluster.

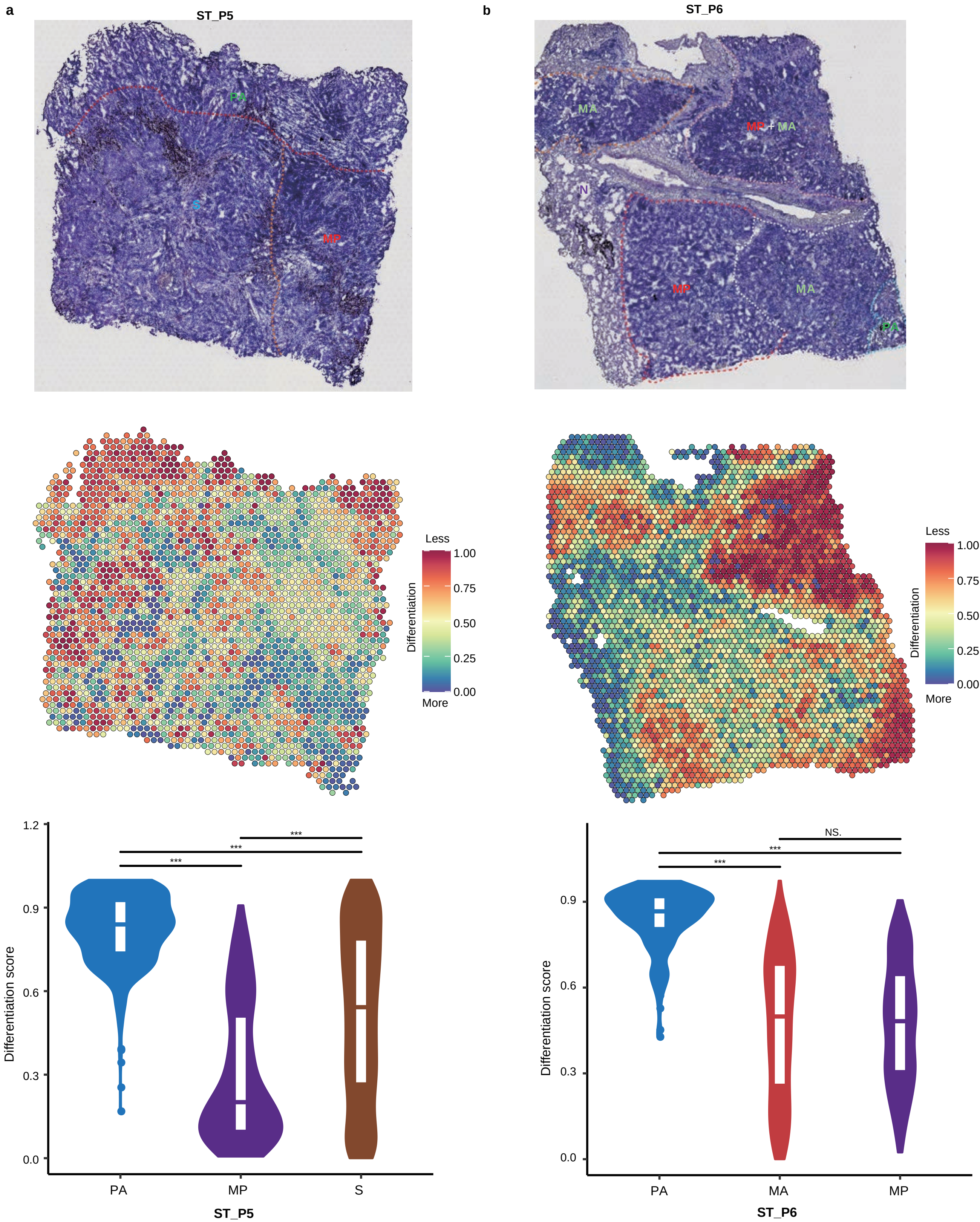

Supplementary Fig. S10 Differentiation states of ST_P5 and ST_P6. Hematoxylin and eosin (H&E) staining, differentiation states of spatial spots, and statistical differences of differentiation states among histologic subtypes in patient **a** ST_P5 and **b** ST_P6.

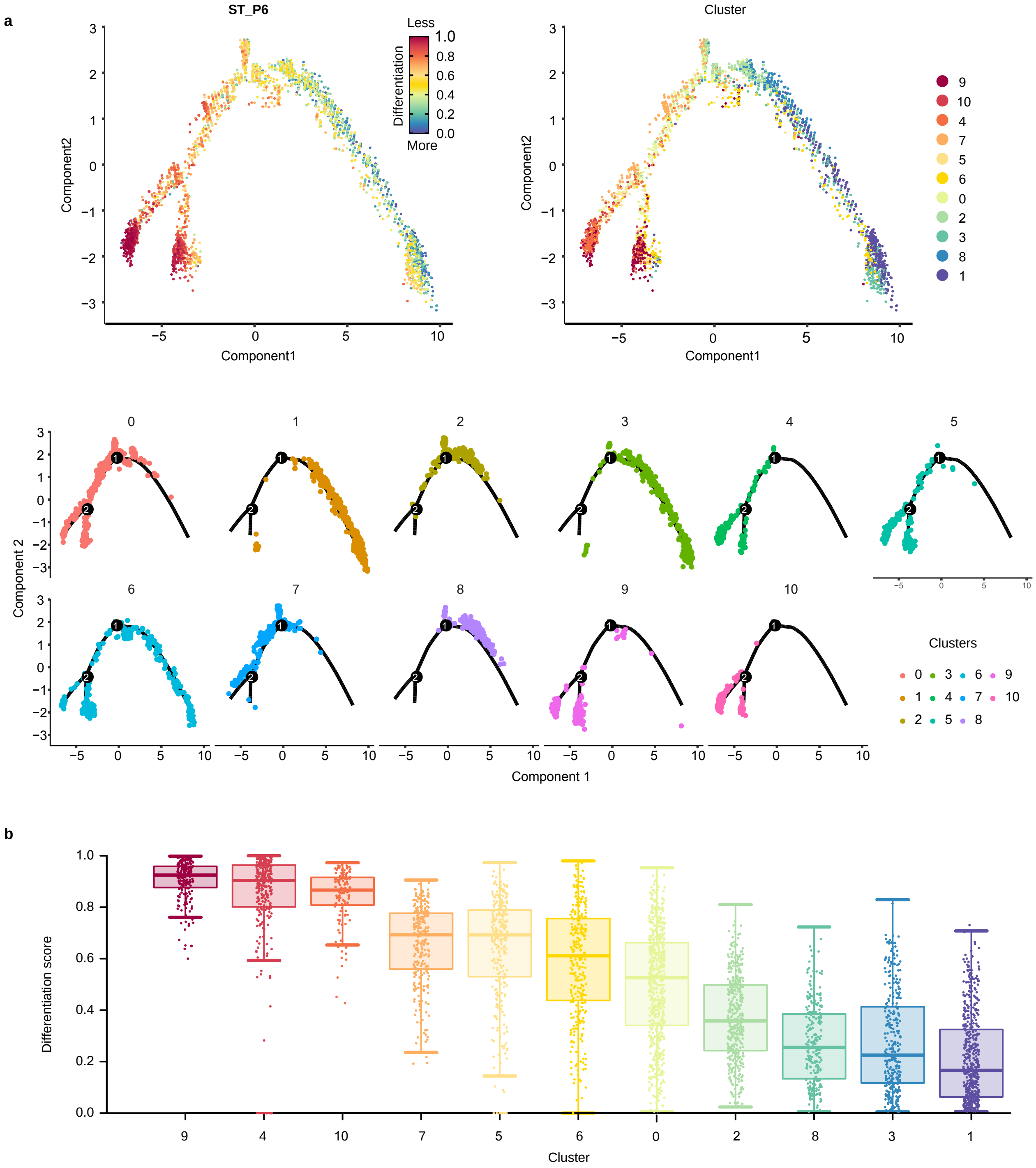

Supplementary Fig. S11 Differentiation trajectories and states of ST_P6. a Differentiated trajectory of each cluster for patient ST_P6 in a two-dimensional space inferred by Monocle2 and CytoTRACE. **b** Boxplots showing the distribution and median of differentiation scores per cluster.

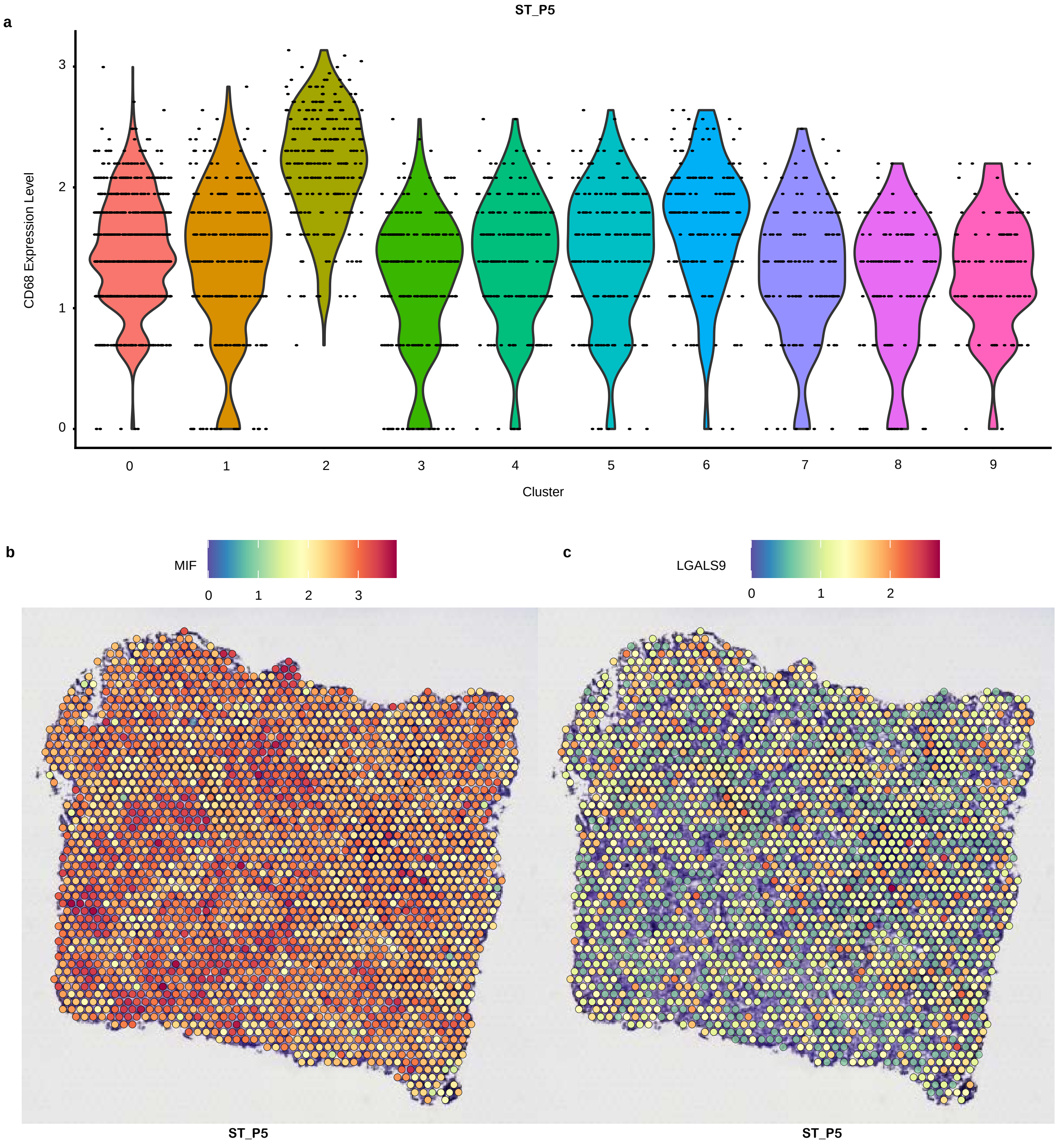

Supplementary Fig. S12 Expression of immune-related genes. a The expression of macrophage marker CD68. Expression of MIF **b** and LGALS9 **c** in histologic section..

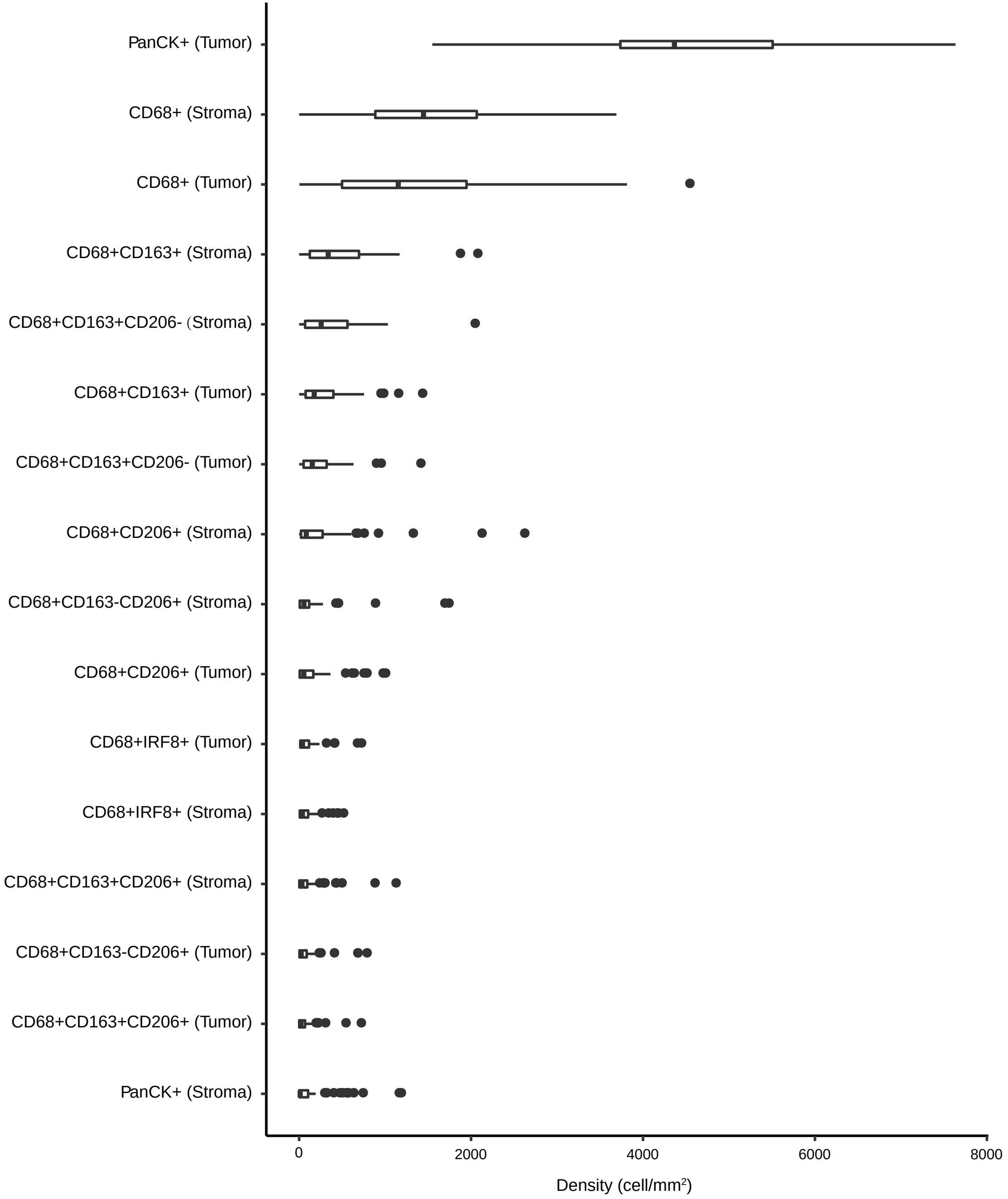

Supplementary Fig. S13 Boxplots showing densities of TAM subpopulations in tumor and stroma regions.

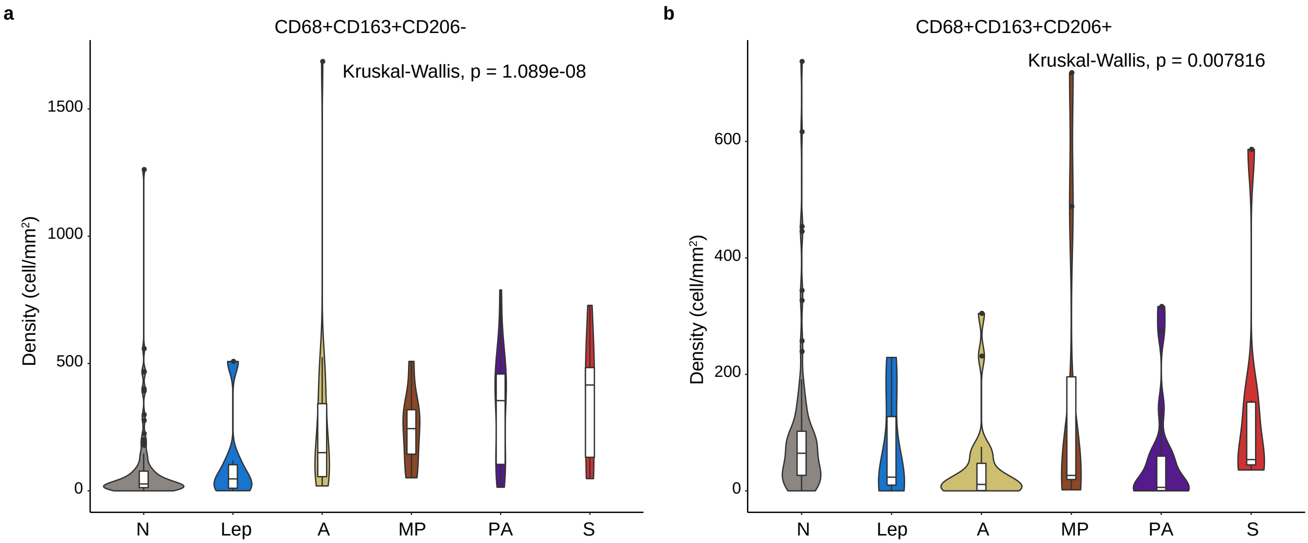

Supplementary Fig. S14 Densities of TAM subpopulations across histologic subtypes. The densities of **a** CD68+CD163+CD206- and **b** CD68+CD163+CD206+ macrophages across histologic subtypes. Normal (N), n = 87; lepidic (Lep), n = 6; acinar (A), n = 22; micropapillary (MP), n = 8; poorly differentiated acinar (PA), n = 17; solid (S), n = 7. Kruskal-Wallis Rank Sum Test.

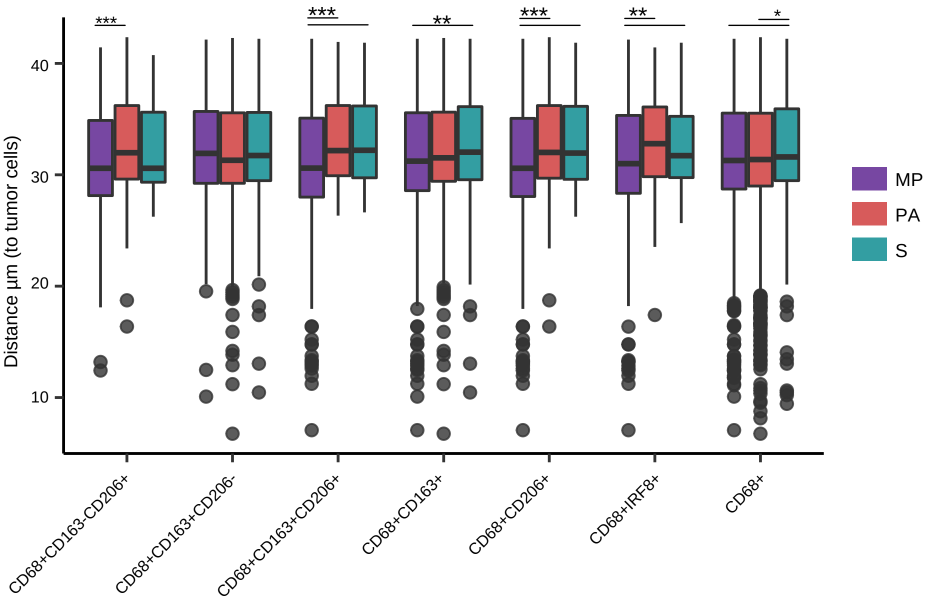

Supplementary Fig. S15 Distance (µm) of TAM subpopulations to tumor cells, and statistical differences among high-grade subtypes.
Kruskal-Wallis Rank Sum Test with Dunn's multiple comparisons post hoc test.

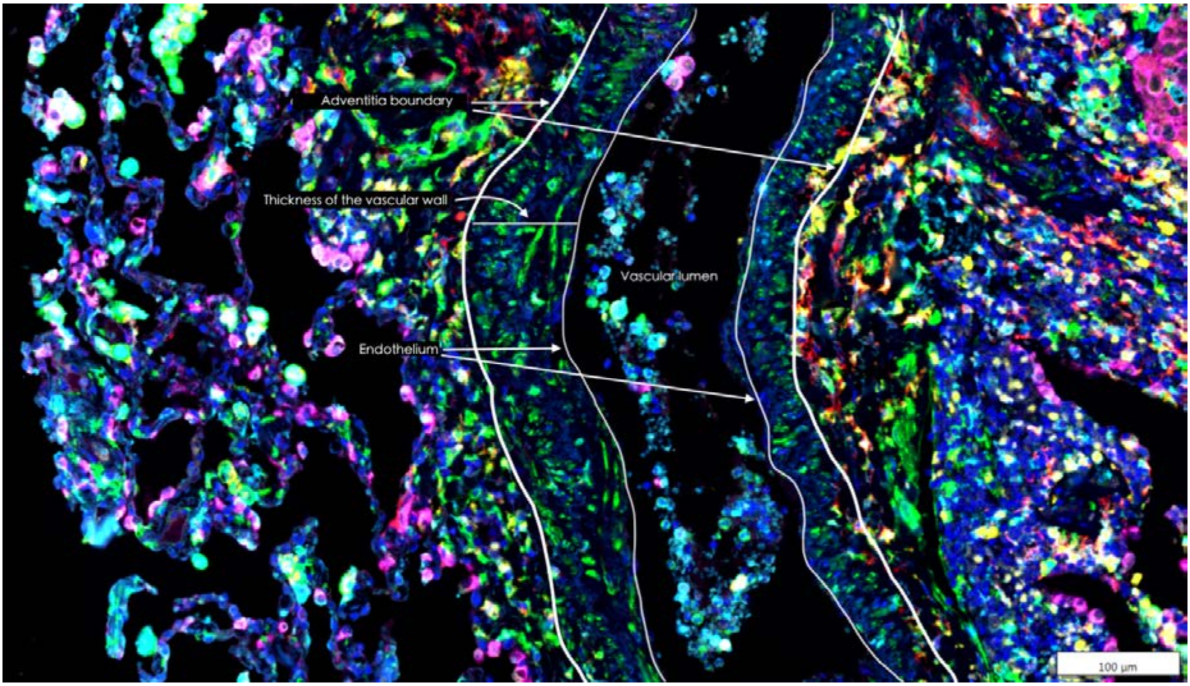

Marker	DAPI	PanCK	CD68	IRF8	CD163	CD206

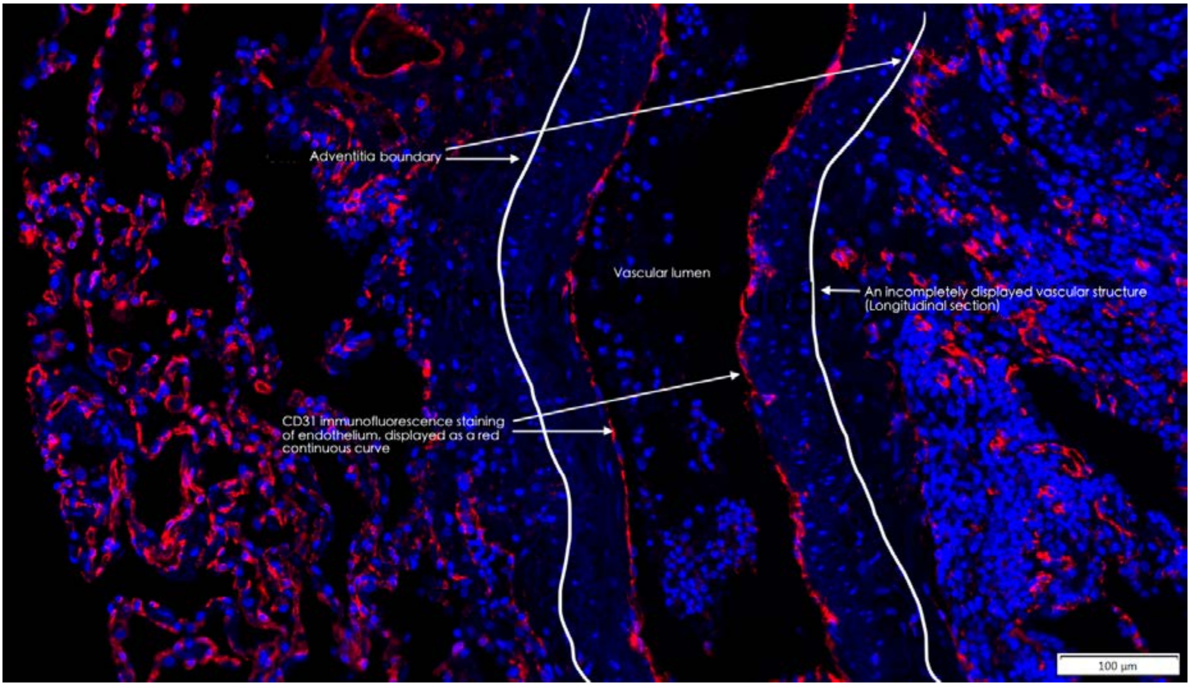

Marker	DAPI	CD31

Supplementary Fig. S16 mIHC images of vessel regions at 10x magnification for Fig. 7d (upper left sample). Top panel, DAPI (blue), PanCK (pink), CD68 (cyan), IRF8 (yellow), CD163 (red), CD206 (green). Bottom panel, we stained vascular marker (CD31) in the adjacent slides of previous tissue microarray (TMA) due to the limitation of channel number in previous mIHC panel. DAPI (blue), CD31 (red). Scale bar, 100 µm.

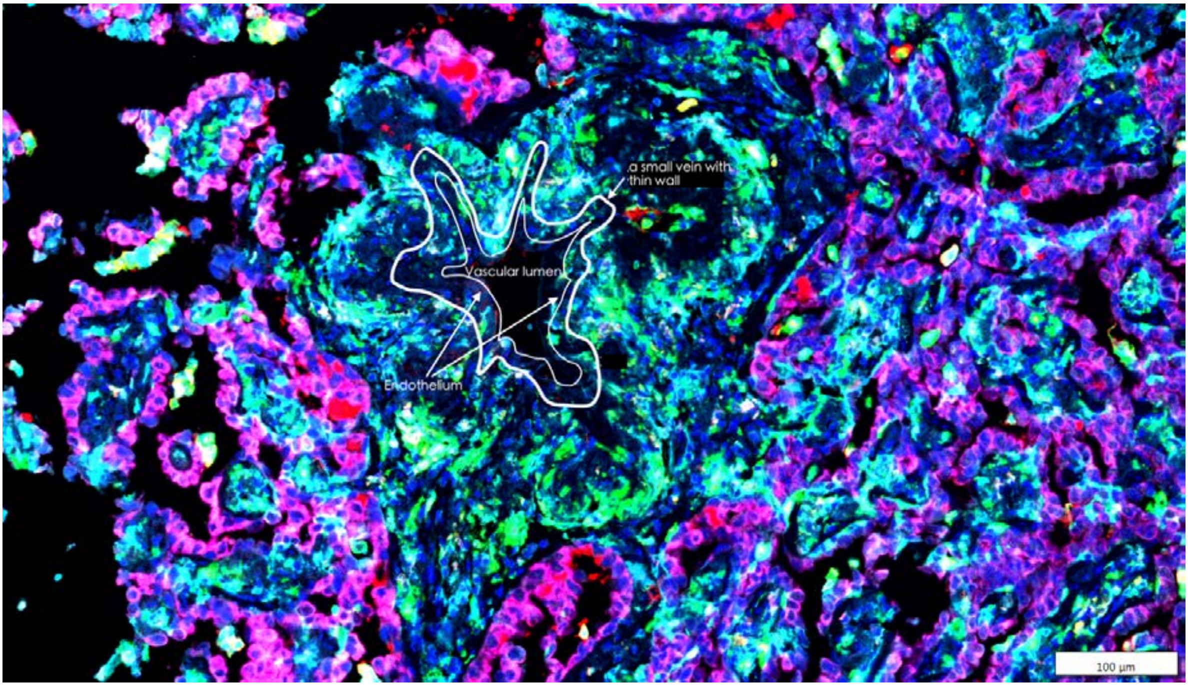

Marker	DAPI	PanCK	CD68	IRF8	CD163	CD206

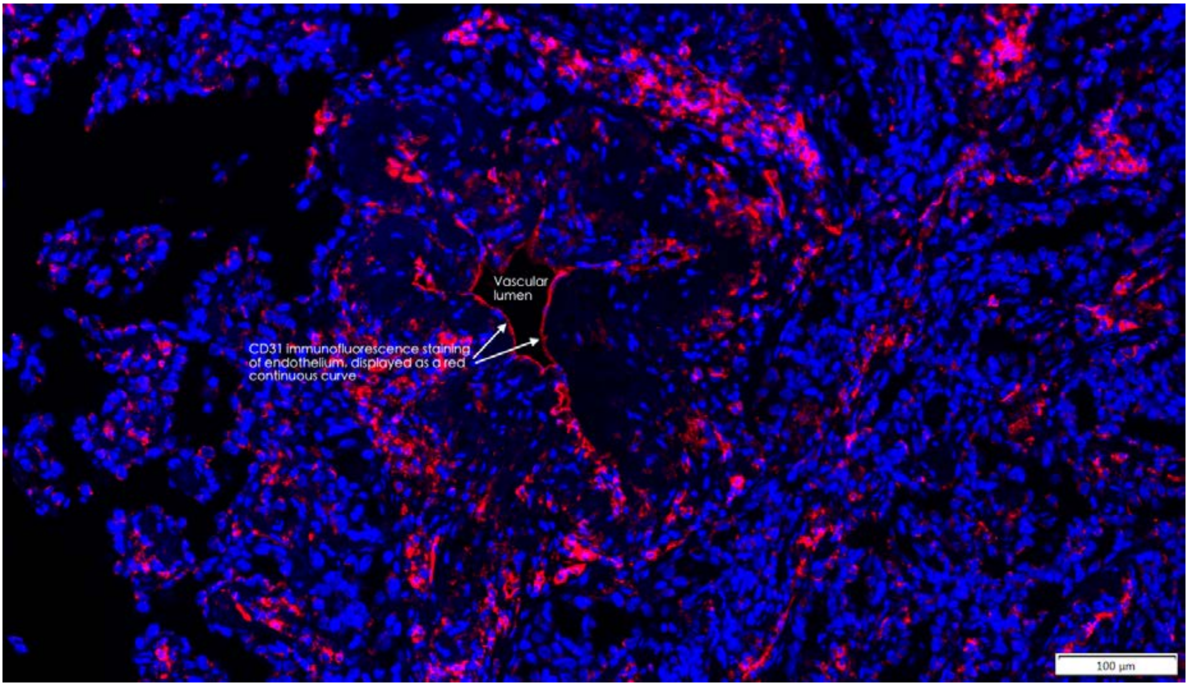

Marker	DAPI	CD31

Supplementary Fig. S17 mIHC images of vessel regions at 10x magnification for Fig. 7d (upper right sample). Top panel, DAPI (blue), PanCK (pink), CD68 (cyan), IRF8 (yellow), CD163 (red), CD206 (green). Bottom panel, we stained vascular marker (CD31) in the adjacent slides of previous tissue microarray (TMA) due to the limitation of channel number in previous mIHC panel. DAPI (blue), CD31 (red). Scale bar, 100 µm.

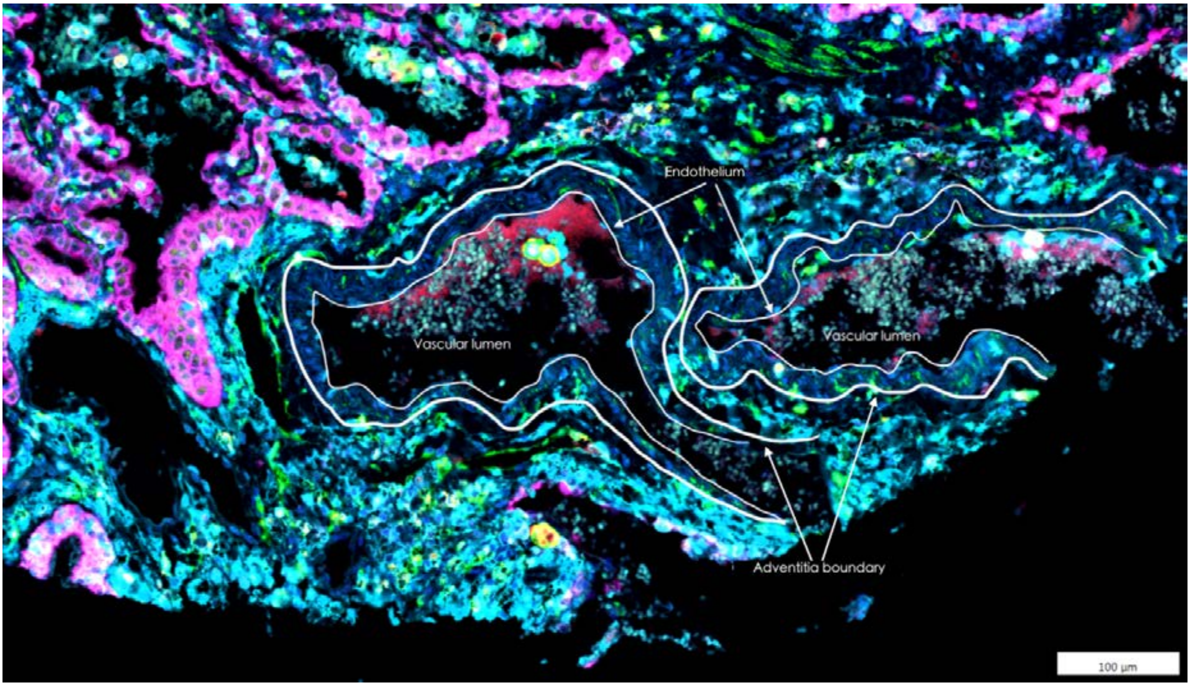

Marker	DAPI	PanCK	CD68	IRF8	CD163	CD206

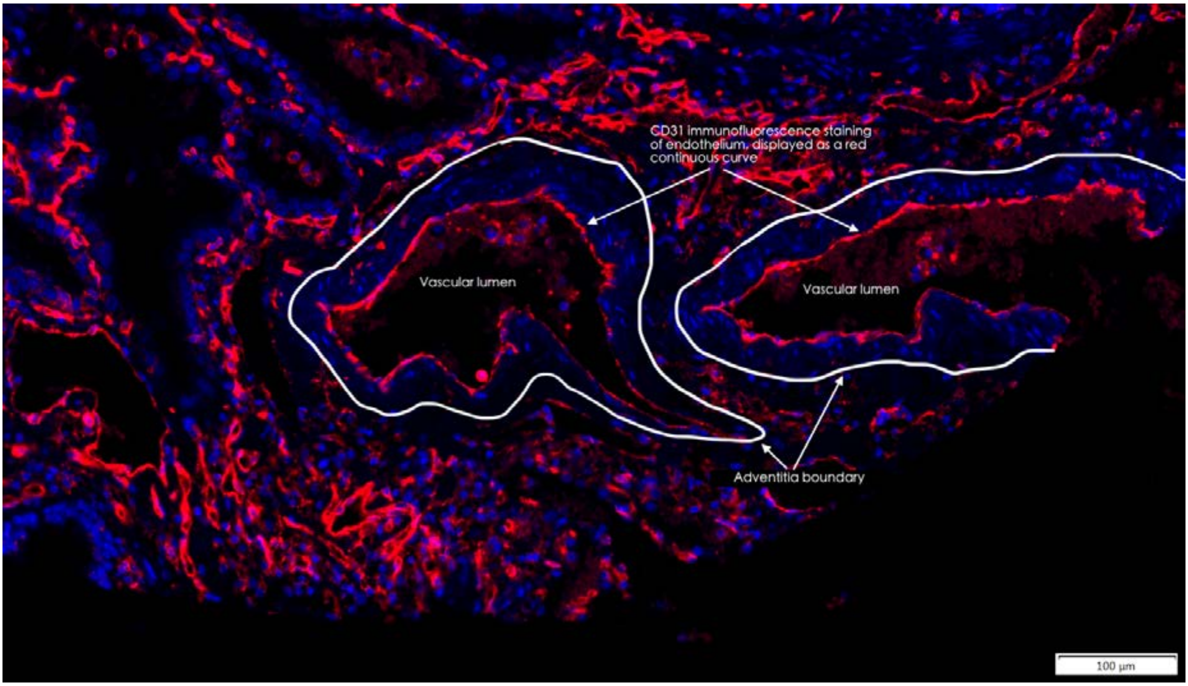

Marker	DAPI	CD31

Supplementary Fig. S18 mIHC images of vessel regions at 10x magnification for Fig. 7d (lower left sample). Top panel, DAPI (blue), PanCK (pink), CD68 (cyan), IRF8 (yellow), CD163 (red), CD206 (green). Bottom panel, we stained vascular marker (CD31) in the adjacent slides of previous tissue microarray (TMA) due to the limitation of channel number in previous mIHC panel. DAPI (blue), CD31 (red). Scale bar, 100 μm.

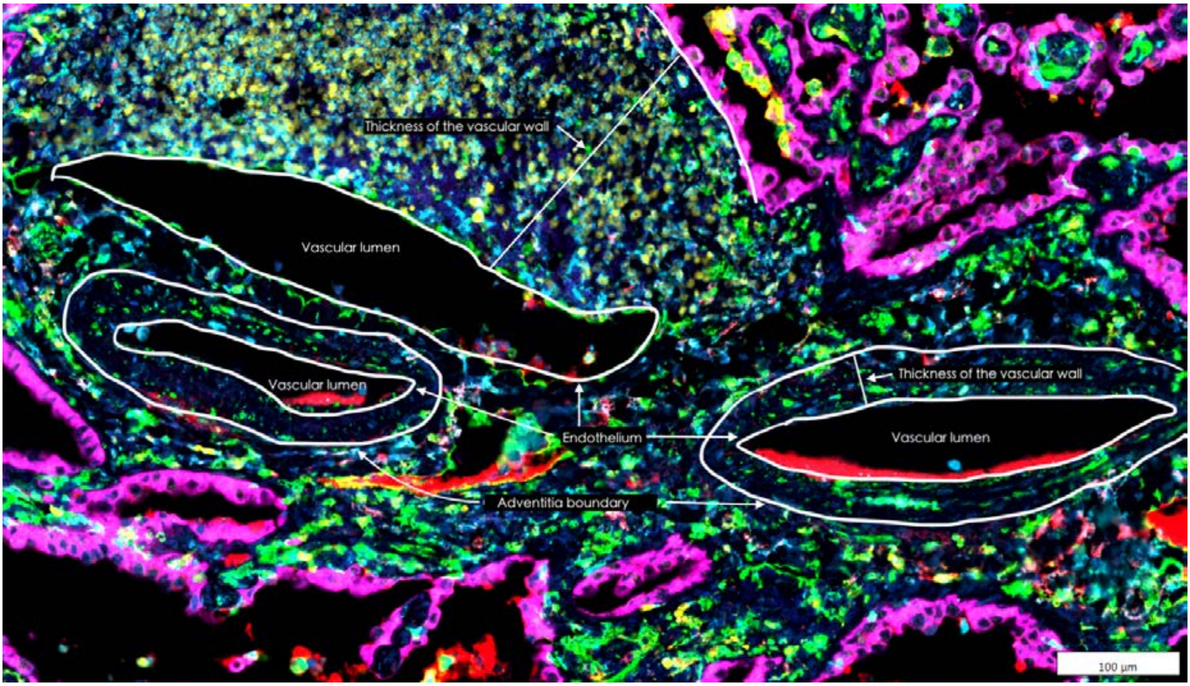

Marker	DAPI	PanCK	CD68	IRF8	CD163	CD206

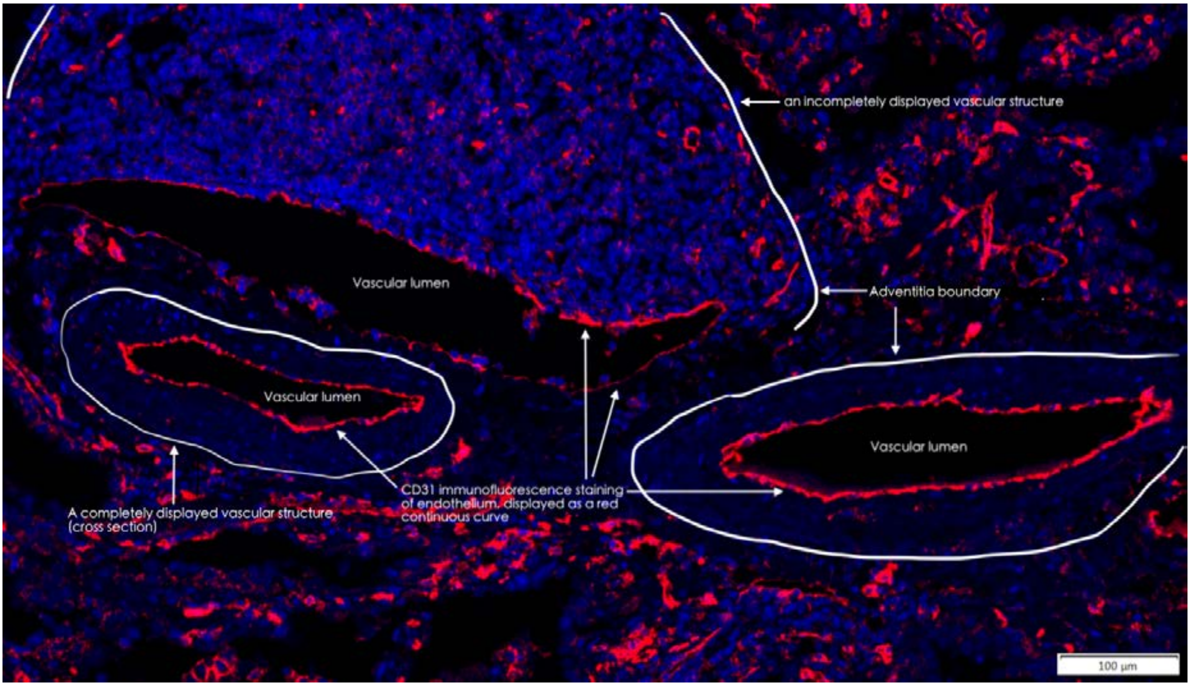

Marker	DAPI	CD31

Supplementary Fig. S19 mIHC images of vessel regions at 10x magnification for Fig. 7d (lower right sample). Top panel, DAPI (blue), PanCK (pink), CD68 (cyan), IRF8 (yellow), CD163 (red), CD206 (green). Bottom panel, we stained vascular marker (CD31) in the adjacent slides of previous tissue microarray (TMA) due to the limitation of channel number in previous mIHC panel. DAPI (blue), CD31 (red). Scale bar, 100 µm.
